# Finite element investigation for improving chest wall reconstruction process using ceramic and polymeric implants

**DOI:** 10.1038/s41598-024-79536-3

**Published:** 2025-01-09

**Authors:** Yomna H. Shash

**Affiliations:** Biomedical Engineering Department, Faculty of Engineering, Helwan University, Cairo, Egypt

**Keywords:** Chest wall reconstruction, Chest implants, Alumina, Zirconia, PEEK, PE, Carbon fiber-reinforced PEEK, Health care, Engineering

## Abstract

Car accidents, infections caused by bacteria or viruses, metastatic lesions, tumors, and malignancies are the most frequent causes of chest wall damage, leading to the removal of the affected area. After excision, artificial bone or synthetic materials are used in chest wall reconstruction to restore the skeletal structure of the chest. Chest implants have traditionally been made from metallic materials like titanium alloys due to their biocompatibility and durability. However, the drawbacks of these materials have prompted researchers to seek alternative materials for use in the reconstruction process. This research aims to explore alternatives to metallic implants in order to overcome their drawbacks and enhance the performance of chest wall reconstruction using the finite element method. In this research, customized implants for the ribs and cartilages are used to repair the defective portion of the chest wall. The implants are made from various materials, including stiff bioceramics (alumina and zirconia), soft polymers (polyether ether ketone (PEEK) and polyethylene (PE)), and polymeric composites (carbon fiber-reinforced PEEK 30 and 60% (CFP 30 and 60%)) as alternatives for titanium. They are tested under normal breathing and impact loading conditions. The null hypothesis suggests that stiff implants will provide optimal results. The results illustrate that when using alumina implants, under normal breathing, the maximum tensile and compressive stresses increased to 11.41 and 15.86 MPa on ribs, while decreasing to 0.32 and 0.324 MPa, and 0.96 and 0.56 Pa on cartilages and lung respectively, compared to titanium. Conversely, when using PE implants, the maximum tensile and compressive stresses decreased to 5.69 and 8.2 MPa on ribs and increased to 0.4 and 0.42 MPa, and 1.71 and 1.1 MPa on cartilages and lung respectively. Under impact force, compared to titanium, the maximum tensile and compressive stresses increased to 47.5 and 49.8 MPa on ribs, and decreased to 1.91 and 6.15 MPa, and 4.56 and 7.7 Pa on cartilages and lung respectively, when using alumina implants. On the other hand, the maximum tensile and compressive stresses decreased to 31 and 23 MPa on ribs and increased to 2.52 and 7.83 MPa, and 5.8 and 9.3 MPa on cartilages and lung respectively, when using PE implants. The highest tensile and compressive strains on ribs were 6,162 and 6,235 µε when using alumina implants under impact force. Additionally, the highest tensile and compressive strains on cartilages and lung were 11,192 and 20,918 µε and 5,836 and 9,335 µε, respectively, when using PE implants. For screws, the peak values of von Mises stress were 61.6 MPa and 433.4 MPa under normal breathing and impact force respectively, when using PE implants. In fatigue analysis, alumina, PEEK, and PE implants failed under impact force as the maximum equivalent alternating stresses exceeded their fatigue limits, resulting in safety factors of less than one. It was concluded that stiff bioceramic implants (alumina and zirconia) produced the lowest stresses and strains on the surrounding cartilages and underlying lung, and the highest stresses and strains on the surrounding ribs, unlike soft PEEK and PE implants. Additionally, CFP 30% and 60% implants distributed stresses on the ribs, cartilages, and lungs similarly to titanium implants. Furthermore, the tensile and compressive stresses and strains on the ribs, cartilages, and lungs did not exceed allowable limits for all used implants. Finally, Zirconia, CFP 30%, and CFP 60% implants can be used as substitutes for titanium in chest wall reconstruction to restore damaged portions of the ribs and cartilage. However, stiff alumina implants and soft PEEK & PE implants were not recommended for use as they were susceptible to fracture under impact force.

## Introduction

The chest wall, also known as the thoracic wall, serves to protect vital organs such as the lungs, liver, and heart. It is composed of bones including the ribs, sternum, and spine, which are connected by cartilage to form a protective chamber in the abdomen^1^. Common causes of chest wall damage include tumors, malignancies, metastatic lesions, bacterial or viral infections, and car accidents. In many cases, portions of the chest wall must be resectioned^2^. Following chest wall resection, artificial bone or metallic implants are used in chest wall reconstruction to help restore the skeletal structure of the chest wall^3,4^.

In chest wall reconstruction, implants and screws are used to restore chest durability^4^. The primary stability of the reconstructed prosthesis is affected by bone quality, as it influences the distribution of loads on all parts^5^. Bone is an organ that can change depending on several factors such as hormones, vitamins, and mechanical influence^5^. Human bones are divided into external cortical bone and internal cancellous bone^6^. Cortical bone is much denser than cancellous bone, making it harder, stronger, and stiffer. From CT scans, the densities of cancellous and cortical bones are nearly 300–700 HU and 1200–3000 HU, respectively, representing normal conditions^7^.

The development of implants has become an essential procedure for successful reconstruction surgery^8^. Implants are often made from metallic materials such as stainless steel, cobalt-based alloys, pure titanium, and titanium alloys (e.g., grade 5)^8,9^. The advantages of metallic materials, such as their great mechanical strength, longevity, and chemical and biological compatibility, are the reason for their selection for the fabrication of implants.

Metals, however, have drawbacks, including the need for surface modification, casting issues, and incompatibility with CT and MRI imaging equipment, which results in radiologic abnormalities^10,11^. Because of their tendency to corrode and wear down, metallic implants can potentially be detrimental as they deposit particles and ions into surrounding tissues^12^. Moreover, metallic implants may cause hypersensitivity reactions such as erythema, urticaria, eczema, edema, pain, and necrosis^13^. They have also been connected to several clinical problems, such as surface degradation and contamination related to peri-implantitis^12,13^. Therefore, efforts have been made to find substitute materials for metallic implants to be utilized in chest wall reconstruction.

When choosing chest implants, several crucial requirements must be met, including biocompatibility and sufficient mechanical properties to tolerate function-related stress^9^. Other important factors to consider when selecting the implant material include cost-effectiveness, production efficiency, speed of preparation, and clinical application readiness.

One feasible option for chest wall reconstruction is to utilize an implant made from stiff bioceramic materials such as alumina or zirconia in place of titanium^14–17^. These ceramics are characterized by their good mechanical properties (durability in compression), good thermal properties (low thermal conductivity), excellent chemical properties (corrosion resistance), biocompatibility, and superior aesthetics^18^. Furthermore, these bioceramics do not cause inflammatory reactions and are robust, nontoxic, and chemically stable in biological environments^19^. Consequently, these bioceramics may provide durable and functional chest implants.

Recently, there has been a lot of interest in the use of polymeric materials in bone repair^20,21^. In cranioplasty, polymeric implants are beneficial in addressing the challenges associated with titanium implants, and they typically do not disrupt therapeutic or diagnostic imaging methods^22^. For rib prostheses, various polymeric materials have been utilized, such as polytetrafluoroethylene, polypropylene mesh, and polymethyl methacrylate and its composites^23,24^.

PEEK is a highly effective polymeric substance that is commonly used in orthopedics for creating implants, plates, fixation screws, and other components for bone fixation and reconstruction^25^. This material exhibits favorable mechanical, chemical, thermal, and electrical properties, as well as radiolucency and biocompatibility in both in vitro and in vivo settings^26^. Unlike metallic materials, PEEK does not cause any clinical issues^27^. It is believed that the material’s low stiffness and excellent shock-absorbing capability could reduce stress-related problems in bones and surrounding tissues^25^.

The addition of carbon, glass fibers, or ceramic fillers at specific percentages improves the mechanical properties of PEEK materials, making them more suitable for bone reconstruction processes^26,28,29^. The loading modulus of PEEK is approximately 3.5 GPa. However, by adding a particular percentage of carbon or glass fibers, a wide range of loading moduli, from 12 to 150 GPa, can be achieved^25,26^. The most commonly used PEEK composites for bone fixation and reconstruction are carbon fiber-reinforced PEEK (30% and 60%)^28^. These composites exhibit strong inertness, biocompatibility, minimal plaque affinity, resistance to chemical erosion, and compatibility with imaging techniques. Additionally, these composites are less expensive and easier to manufacture than metallic and ceramic materials^28,29^.

Polyethylene, another soft polymeric substance, has been used in joint replacement and bone repair^30^. It has been shown that polyethylene is a chemically inert material with low abrasion, impact, and tissue reactivity, as well as lubricity^31^. Therefore, this substance can be used in implants as a replacement for bone or cartilage. However, further research is required to confirm its potential use in reconstructing damaged ribs and cartilage, as well as to assess its effects on surrounding structures and underlying lung tissue.

The finite element method (FEM) has been instrumental in solving various biomedical problems and evaluating a design’s performance under practical conditions. It is particularly beneficial in studying biomechanical properties and predicting the body’s response to different mechanical configurations^32^. In the fields of maxillofacial surgery and bone reconstruction, FEM plays a crucial role in treatment planning, implant and instrument design, and the creation of anatomically accurate prostheses^32,33^. FEM can accurately replicate complex geometries, correct flaws, propose alternative designs, simulate different materials under varying conditions, and analyze internal stresses and strains throughout the chest wall reconstruction process while assessing implant materials^34–36^.

Previous studies have utilized the finite element method in the fixation and reconstruction of the chest wall^37–40^. In Zhang et al..‘s research^38^, the biomechanics of carbon fiber artificial ribs were evaluated using finite element analysis and clinical implementation. In Bauman et al..‘s work^37^, finite element analysis was used to evaluate the stability of chest wall injuries after surgical stabilization of all rib fractures. By utilizing the finite element method, the biomechanical behavior in chest compression for rigid and flexible implants was analyzed and evaluated^39^. In Xie et al..‘s study^40^, a novel method to individually design and optimize the shape of the Nuss bar was constructed using CT imaging data of the patient’s thorax. The proposed method involved constructing a three-dimensional model of the human thorax using CT images of a patient with a funnel chest. Then, a finite element model was constructed in which different operative plans were simulated.

Several experimental studies have been conducted to determine the material properties and force-deformation behaviors of rib tissues under bending or tensile loading^41,42^. However, further research is necessary to provide biomechanical analysis of chest wall prostheses in terms of mechanical resistance and to evaluate the effect of changing the prosthetic implant material on the surrounding structure and the performance of the reconstruction process under physiological and impact loads. Therefore, this research aims to enhance the chest wall reconstruction process by replacing metals with ceramics and polymers in the fabrication of implants for reconstructing resected portions of ribs and cartilages. The mechanical performance of these implants will be evaluated using the finite element method (FEM).

The materials used for the implants include stiff bioceramics (alumina and zirconia), soft polymers (polyether ether ketone (PEEK) and polyethylene (PE)), and polymeric composites (carbon fiber-reinforced PEEK 30 and 60% (CFP 30 and 60%)). Stress-strain analyses are conducted on the ribs, cartilages, and lungs to determine the most suitable reconstruction material for the resected portions of the ribs and cartilages with varying characteristics, and to assess its impact on the surrounding structures. This study examines two distinct loading scenarios: normal breathing and impact force, in addition to a fatigue study on implants. The null hypothesis posits that stiff implants will offer optimal results when used in the reconstruction of both ribs and cartilages, especially under impact force.

## Methodology

The finite element model was constructed and stimulated by the following steps:

### First step: model generation and chest wall reconstruction

**Fig. 1:**
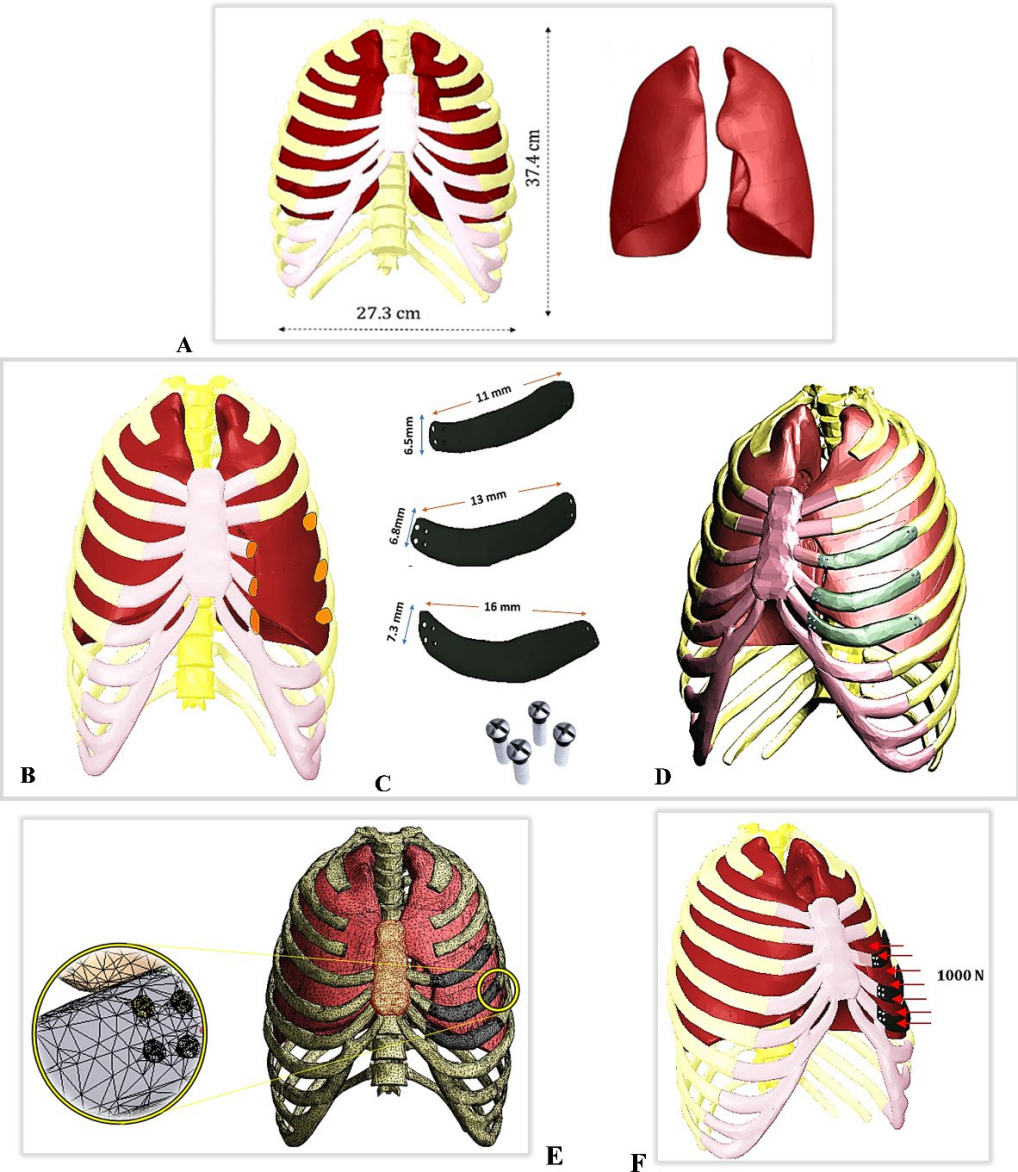
FEM model: (**A**) Intact rib cage with lungs, (**B**) Defective chest wall, (**C**) Custom- made implants and screws, (**D**) Final model after chest wall reconstruction process, (**E**) Meshing, and (**F**) Impact force.

The complex healthy (intact) model of the human chest, including the sternum, 24 ribs, cartilages, 12 thoracic vertebrae, and 2 lungs, was downloaded from the anatomical database “BodyParts3D/Anatomography^43^” as OBJ files (Fig. 1A). These files were created based on computerized tomography (CT) images. The CT images in this database were taken every 2 mm from the top of the head to the feet of a 22-year-old volunteer with a body mass index of 21.7^44^. During the image acquisition, the individual was in a supine position with hands on the sides of the body, and feet and ankles positioned as if standing.

The OBJ files of the human chest were exported to “Space Claim” software for repair, modification, and solidification. The initial step in the repair process involved checking the facets and automatically fixing them, followed by cleaning up any holes, intersections, and sharp edges. After the solidification procedures were completed, the second repair step was initiated. Repair tools such as solidify, fix, fix curves, and adjust were utilized to simplify complex faces and curves, merge faces, remove small faces, and fix any bad faces, missing faces, curves, or gaps^45^.

To replicate an unhealthy case, large defects were created in the left fourth, fifth, and sixth ribs and connected cartilages that require resection (Fig. 1B). Artificial implants were utilized to replace the damaged sections of the chest in cases where injuries from accidents or other trauma prevent certain ribs and cartilages from healing^3,4^. For the design of the implants and to simulate a realistic scenario, the chest model was assumed to be symmetrical along its mid-sagittal plane, and the resected left portion was mirrored from the right side. The lengths of the first, second, and third implants were approximately 11, 13, and 16 mm, respectively, while the average thicknesses were 6.5, 6.8, and 7.3 mm, as shown in Fig. 1C. Figure 1D illustrates the process of reconstructing the defective chest using three customized implants. In practice, the implants were typically connected to the surrounding structures using medical sutures or screws^46,47^. In this model, each implant was connected to the surrounding rib and cartilage by four screws on each side. Therefore, a total of 24 screws were used, with each screw having a radius of 1 mm and a length of 11 mm.

The implants and screws were assumed to be in bonded connections with the surrounding ribs and cartilages to stimulate complete osseointegration, with no motion under the application of internal and external loads. At the end of this stage, the geometries were assembled and exported to “ANSYS” software (ANSYS 18.1, Houston, Texas, USA) to perform all calculations.

### Second step: materials selection

In this research, conventional materials (titanium), stiff ceramics (alumina & zirconia), soft polymers (PEEK & PE), and polymeric composites with carbon fibers (carbon fiber-reinforced PEEK 30 & 60%) were used as implants to reconstruct defective portions of ribs and cartilages. Additionally, all fixation screws were made of titanium. The properties of the materials used in this study, such as loading modulus, poisson’s ratio, density, and yield strength, are listed in Table 1.

Ribs, cartilage, and lungs have non-linear characteristics, so their material properties vary in different directions. Forman et al.^47^ used a linear isotropic elastic model for the bulk cartilaginous tissue along with a hyper-elastic non-isotropic constitutive model. The results showed that the linear model, commonly used in numerical simulations of the ribcage, adequately represents the mechanical behavior of the cartilage.

The breastbone and osseous portion of the ribs have a complex structure consisting of external cortical bone [1200–3000 HU] and internal cancellous bone [300–700 HU], making their mechanical properties anisotropic. Previous studies^48–50^ have shown that a map of the bone’s anisotropic directions can be determined from CT scans with high image resolution (a few tens of a µm). However, obtaining micro-CT scans of an adult ribcage involves high radiation levels, usually only possible post-mortem. As a result, previous studies^46,51–53^ have often used isotropic properties for ribs. In Zhu et al.‘s research^54^, the relationship between the mechanical properties of human ribs and the density of bone was measured using high-resolution quantitative computed tomography. The results showed that the average density of human ribs is 0.736 g/cm3.

The influences of sex, loading rate, and age on the tensile material properties of human rib cortical bone were evaluated in the research by Katzenberger et al.^55^. In this study, sixty-one participants ranging in age from 17 to 99 were included. Each participant provided two rectangular coupons of rib cortical bone, which were then milled for testing. A material testing device was used to test one coupon for failure in tension at a specific strain rate of 0.005 strain/s and another coupon at 0.5 strain/s for each participant. Axial load was measured using a reaction load cell, and displacement was measured using an extensometer. Subsequently, the elastic strain energy density (SED), plastic SED, total SED, yield stress, yield strain, failure stress, failure strain, ultimate stress, and elastic modulus were computed for each test. The results showed that human ribs have a loading modulus of 10 to 17 GPa, a yield strength of 60 to 100 MPa, and an ultimate tensile strength of 80 to 120 MPa.

In the study by Albert et al.^56^. , novel techniques were developed to measure the material properties of human rib under compression and compare the compressive material property data to the tensile data for matched participants. Cylindrical coupons were obtained from the rib cortical bone of 30 participants, with each participant providing two coupons. Compression tests were performed on one coupon at a rate of 0.005 strain/s and on another at a rate of 0.5 strain/s. The elastic modulus, yield stress, yield strain, ultimate stress, ultimate strain, elastic strain energy density (SED), plastic SED, and total SED were computed by recording load and displacement data. The yield and ultimate compressive strengths were found to be approximately 90 to 120 MPa and 180 to 200 MPa, respectively.

According to studies^57,58^, the lung can be considered an isotropic substance because its properties do not depend on specific preferential directions. Therefore, to simplify the analysis and reduce calculation time, linear characteristics were assumed, and material properties were applied based on previous studies^52,53,59–62^.

**Table 1:** The properties of the ribs, lungs, cartilage and implants.

	Loading Modulus (MPa)	Poisson’s Ratio	Density (kg/m3)	Tensile Yield Strength (MPa)	Compressive Yield Strength (MPa)	Ref
Lungs	1e-3	0.30	394	0.3e-3	0.8e-3	^59,60^
Ribs	15,000	0.30	736	60–100	90–120	^52,53^
Cartilage	24.5	0.40	1000	5.6	8.3	^61,62^
Implants						
Titanium	110,000	0.34	4500	862	848	^63^
Alumina	380,000	0.45	3950	275	2600	^64^
Zirconia	210,000	0.45	6000	1200	2500	^65^
PEEK	3,500	0.40	1300	140	140	^26^
CFP 60%	150,000	0.35	1600	2000	800	^26,28^
CFP 30%	18,000	0.35	1370	200	300	^26,28^
PE	1,500	0.4	995	23	43	^66^

CFP: Carbon Fiber Reinforced PEEK.

PE: Polyethylene.

PEEK: Poly ether ether ketone.

### Third step: mesh adjustment

**Fig. 2:**
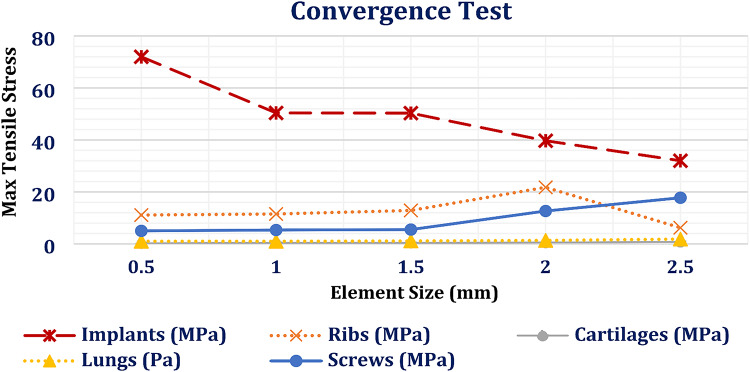
Convergence test: The relation between element size (mm) and the maximum tensile stresses on implants, screws, ribs, lungs and cartilages, using titanium implants, under normal breathing.

The mesh division method plays a crucial role in enhancing the accuracy of the results obtained from the finite element method^45^. In this study, an “Adaptive” size function was utilized with a tetrahedral mesh and element sizes ranging from 0.5 to 1 mm in the ANSYS program, resulting in a large mesh comprised of elements and nodes (Fig. 1E; Table 2). Mesh refinement was determined through a convergence test. Figure 2 shows the relationship between element size (ranging from 0.5 to 2.5 mm) and the maximum tensile stresses experienced by titanium implants, ribs, cartilages, and lungs under normal breathing conditions. In order to maintain consistency throughout the finite element analyses, the same mesh settings were applied for the impact loading scenario.

**Table 2:** The number of elements and nodes of model parts.

	No. of Elements	No. of Nodes
Lungs	825,544	11,149,837
Ribs	129,013	1,115,334
Cartilages & Sternum	56,665	113,459
Implants	18,689	65,645
Screws	226,564	9,155,843

### Fourth step: loads and constraints

During inhalation, the diaphragm descends, the volume of the pleural cavity expands, the chest expands, and the chest pressure decreases in a typical normal breathing pattern^67^. During exhalation, the process is reversed, resulting in a decrease in the volume of the pleural cavity and an increase in chest pressure^67^. Therefore, for normal breathing analysis, the extreme case of maximum thoracic pressure (40 KPa) was applied to the sternum and interior surfaces of the ribs and cartilage, according to^51,68^. To simulate the impact during a car accident, an additional force of 1000 N was applied and distributed on the three implants as depicted in Fig. 1F^68^. In terms of boundary conditions, the rib-vertebrae connections were constrained to prevent the model from shifting or moving when pressure and force applied^41,63^.

### Final step: results extraction

In the ANSYS program, the endurance of implants with various materials in static analysis was evaluated by extracting the maximum tensile stresses (peak maximum principal stresses) and maximum compressive stresses (peak negative minimum principal stresses). These values were then compared to the tensile and compressive yield limits based on the principal stress theory (Rankine theory)^69^, as well as previous studies^70,71^. According to the Rankine theory, failure occurs when the maximum tensile stress in the complex system reaches the yield limit value in the tension test or when the maximum compressive stress reaches the yield limit value in the compression test^69^. Fatigue analysis was also applied to the three customized implants with different materials under normal breathing and impact force to evaluate their failures or endurances after a certain number of repeated loading and unloading.

For titanium screws, the maximum von Mises stresses were extracted and compared to the yield strength of titanium (approximately 850 MPa) due to its ductile properties, as outlined by the von Mises Yield Criterion^69^. Because ductile materials have similar tensile and compressive strengths, extracting the maximum von Mises stress is an effective way to evaluate their endurance.

For biological components, the maximum tensile and compressive stresses were determined for ribs, cartilages, and lungs in order to assess their responses by comparing them to the tensile and compressive yield limits, as shown in Table 1. Additionally, the estimated tensile and compressive strains were compared to allowable limits due to the potential for tissue microdamage resulting from the concentration and distribution of excessive stresses. Rib damage occurred when the strain exceeded the allowable limits of 20,000 and 25,000 µε in tension and compression^53^. The tensile and compressive limits were approximately 120,000 and 150,000 µε for cartilage, respectively, and 250,000-300,000 µε for lungs^59,61^.

## Results

### Intact model (validation study)

**Fig. 3:**
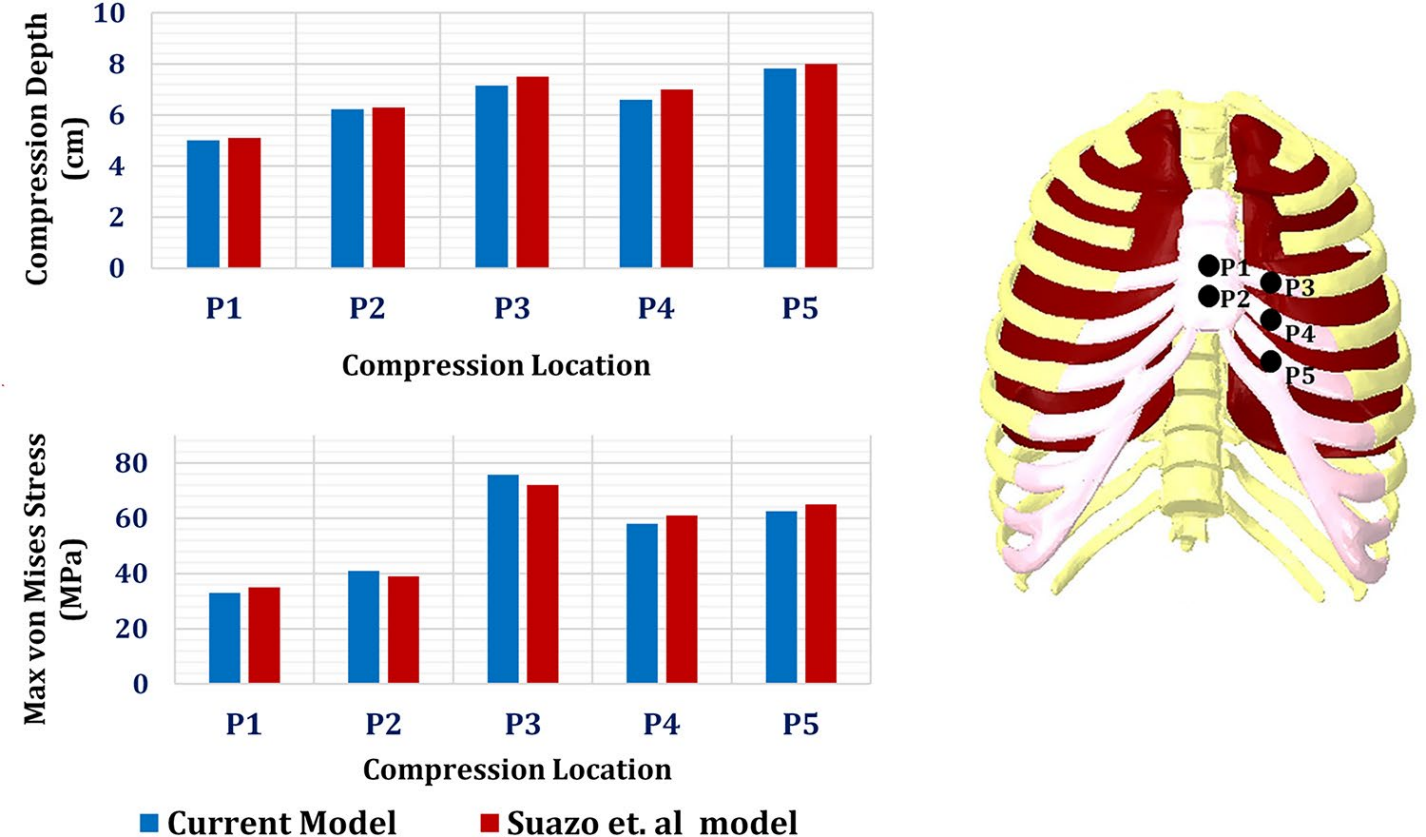
Comparison between the current model and Suazo et al. model^72^: (**A**) Compression depth (cm) and (**B**) Maximum von Mises stress (MPa).

The intact model of the rib cage was validated using Suazo et al..‘s^72^ model. The rib cage model was created based on the computerized tomography (CT) images found in the BodyParts3D database for anatomy^43,44^. Five compression areas (P1, P2, P3, P4, and P5) were included in the simulations to mimic cardiopulmonary resuscitation maneuvers. Each compression region was defined as a surface patch with an area of 10 cm^2^. The compression area P1 and P2 were positioned at the center of the breastbone midline and below the center respectively, while the other three compression areas, P3, P4, and P5, were slightly offset to the cartilaginous tissue of the 4th, 5th, and 6th left ribs, respectively. A force of 600 N was applied to the compression areas, and the maximum compression depth (cm) and maximum von Mises stresses (MPa) were recorded for the rib cage, as shown in Fig. 3.

Compared to the results of Suazo et al.^72^, the compression depth extracted from the current model decreased by 1.76%, 1.26%, 4.8%, 5.85%, and 2.25% using the five compression areas P1, P2, P3, P4, and P5, respectively. Additionally, the maximum von Mises stress changed by -6%, 5.12%, 5.25%, -4.93%, and − 3.78% using the five compression areas, respectively.

### Normal breathing

#### Stresses on implants

**Fig. 4:**
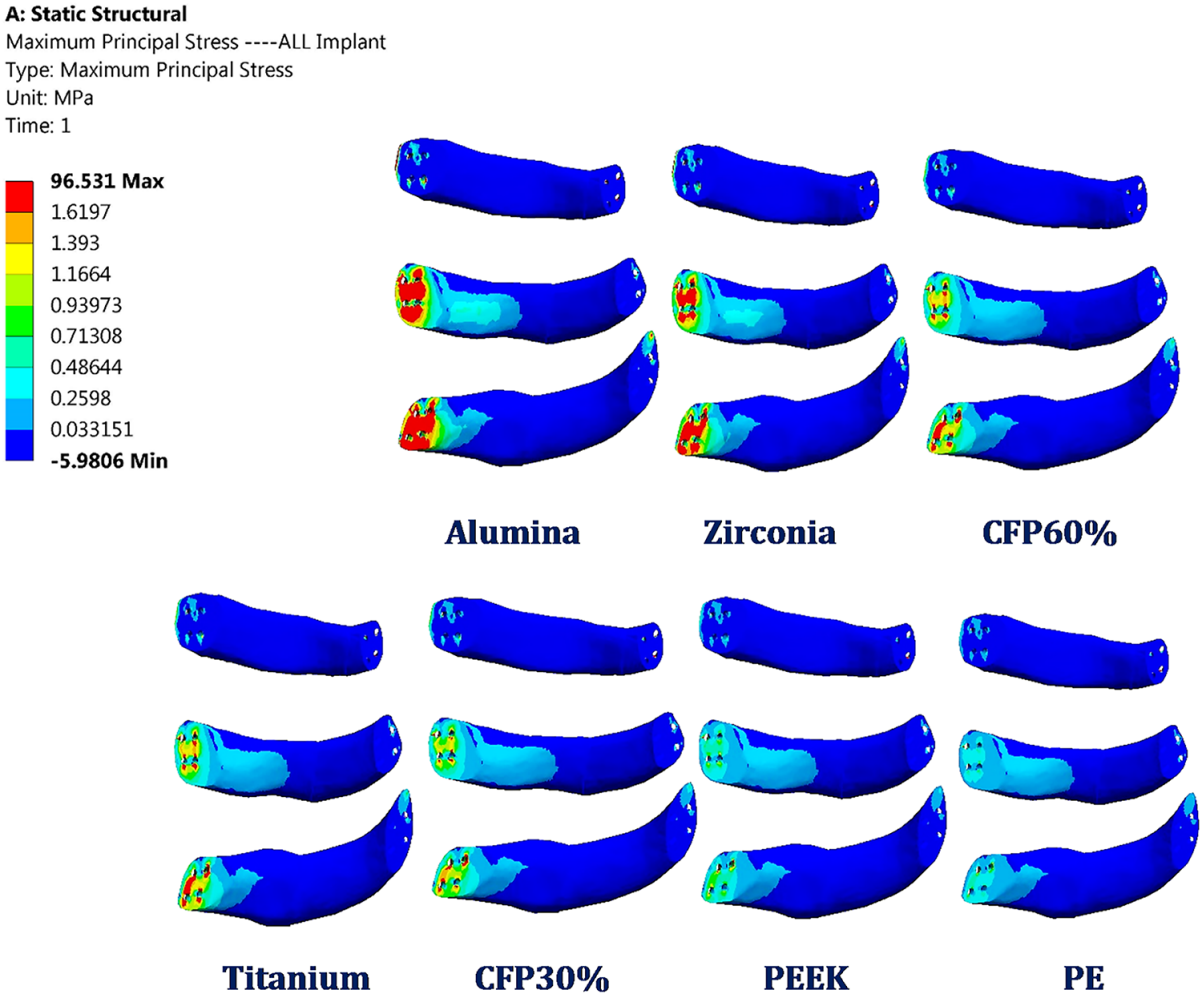
Distribution of maximum principal stresses (MPa) on the alumina, zirconia, CFP 60%, titanium, CFP 30%, PEEK and PE implants (back view) under normal breathing.

The maximum tensile and compressive stresses were extracted in Table 3 to compare implants with their yield strengths and evaluate their endurance under normal breathing conditions. Compared to titanium implants, the maximum tensile and compressive stresses increased by 91.34% and 78.08%, 39.88% and 35.85%, and 22.27% and 19.09% on alumina, zirconia, and CFP 60% implants, respectively. The maximum tensile and compressive stresses reduced by 16.10% and 18.99%, 30.42% and 33.91%, and 69.73% and 73.43% on CFP 30%, PEEK, and PE implants. Figure 4 illustrates the distribution of maximum principal stresses on all implants under normal breathing conditions. The maximum tensile stresses were observed in the back of implants at the contact areas with ribs through the screws.

**Table 3:** Maximum tensile and compressive stresses (MPa) on implants under normal breathing.

	AL	ZR	CFP60	TI	CFP30	PEEK	PE
Implants							
Max Tensile Stress	96.531	70.571	61.686	50.45	42.33	35.104	15.273
Max Compressive Stress	-86.921	-66.31	-58.129	-48.81	-39.54	-32.257	-12.97

#### Stresses on screws

**Fig. 5:**
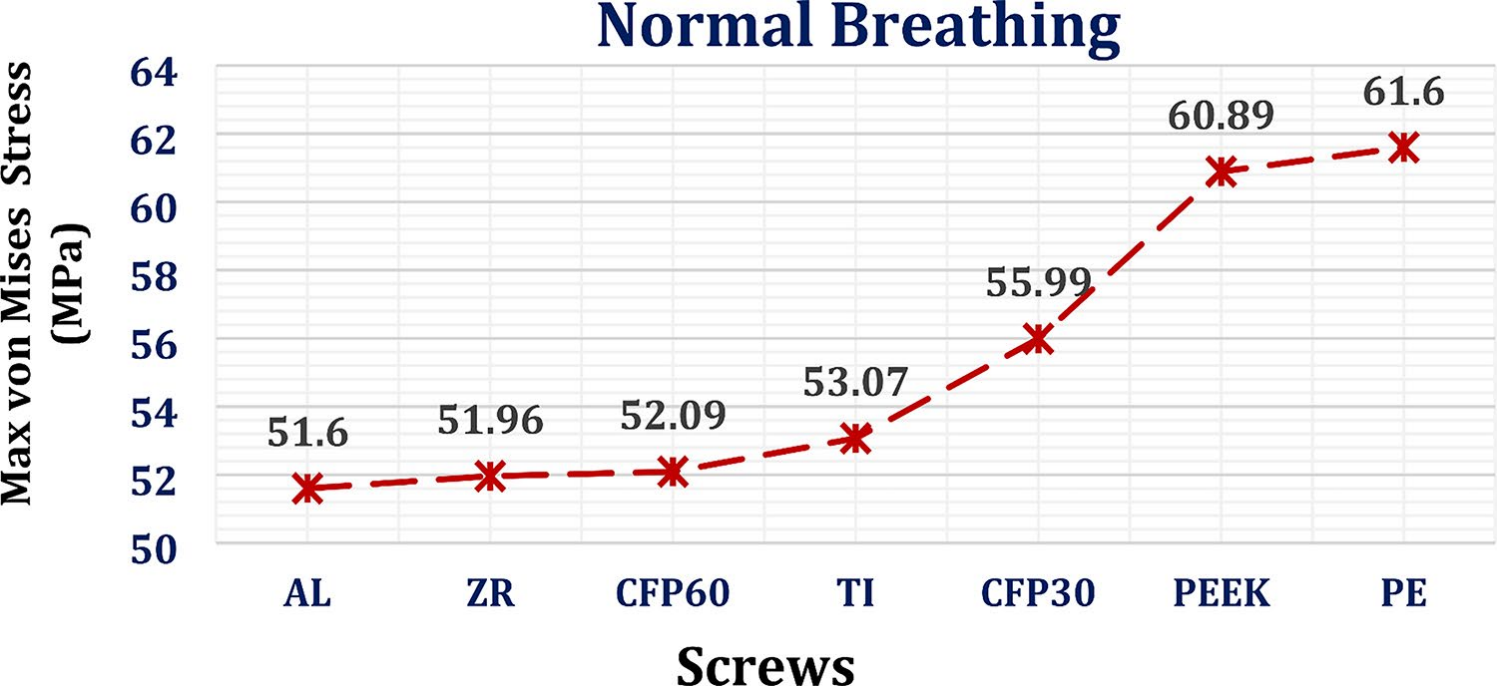
Maximum von Mises stress (MPa) on screws, using different implants materials, under normal breathing.

For screws, the maximum von Mises stresses were extracted (Fig. 5) to check their endurance and compared to the titanium limit of 850 MPa, due to its ductility, according to the von Mises Yield Criterion^69^. When compared with titanium implants, alumina, zirconia, and CFP 60% implants decreased the maximum von Mises stress on the screws by 2.77%, 2.09%, and 1.84%, respectively, under normal breathing conditions. In contrast, the CFP 30%, PEEK, and PE implants increased the maximum stress on the screws by 5.5%, 14.73%, and 16.07%, respectively.

#### Stresses and strains on biological parts

**Fig. 6:**
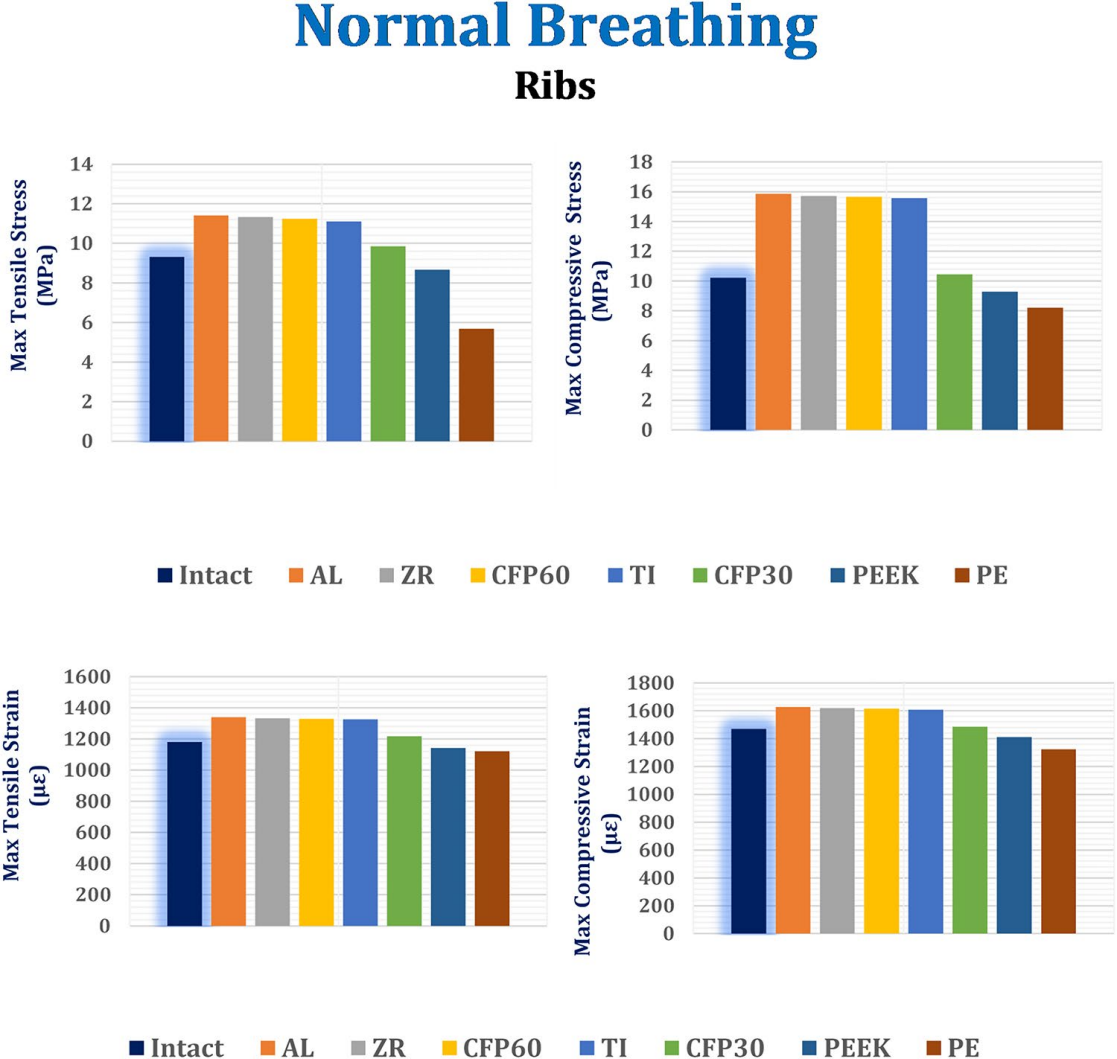
Maximum tensile and compressive stresses (MPa) and maximum tensile and compressive strains (µε) on the ribs under normal breathing.

**Fig. 7:**
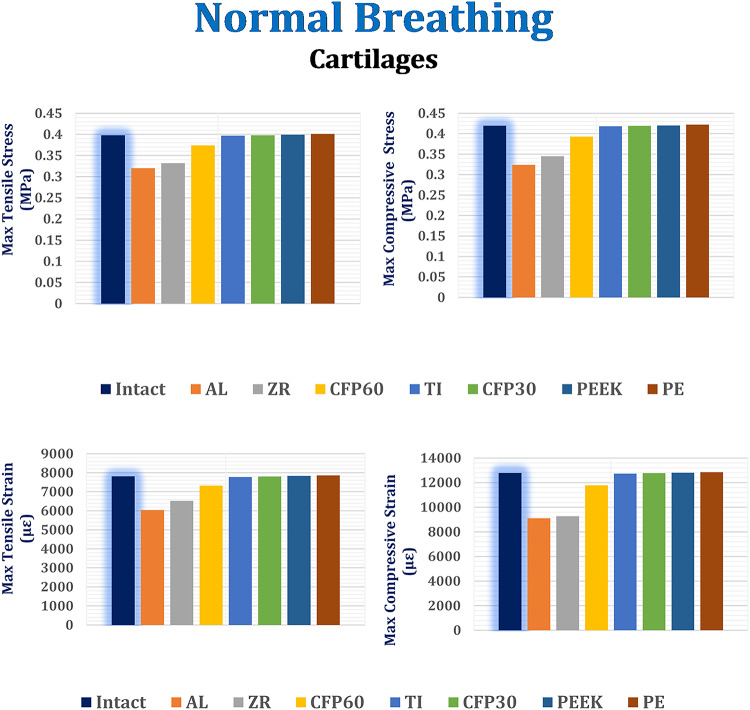
Maximum tensile and compressive stresses (MPa) and maximum tensile and compressive strains (µε) on the cartilages under normal breathing.

**Fig. 8:**
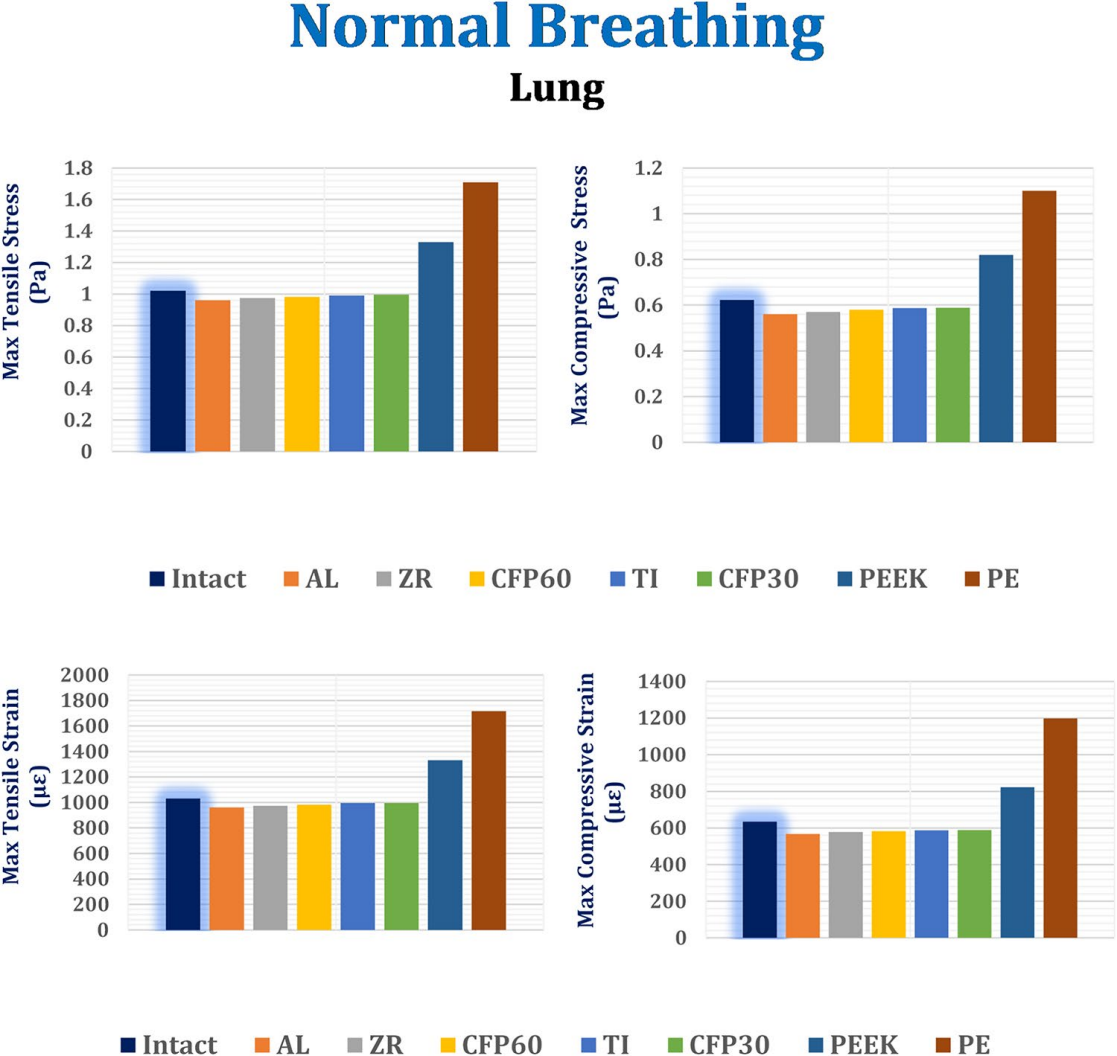
Maximum tensile and compressive stresses (Pa) and maximum tensile and compressive strains (µε) on the left lung under normal breathing.

**Fig. 9:**
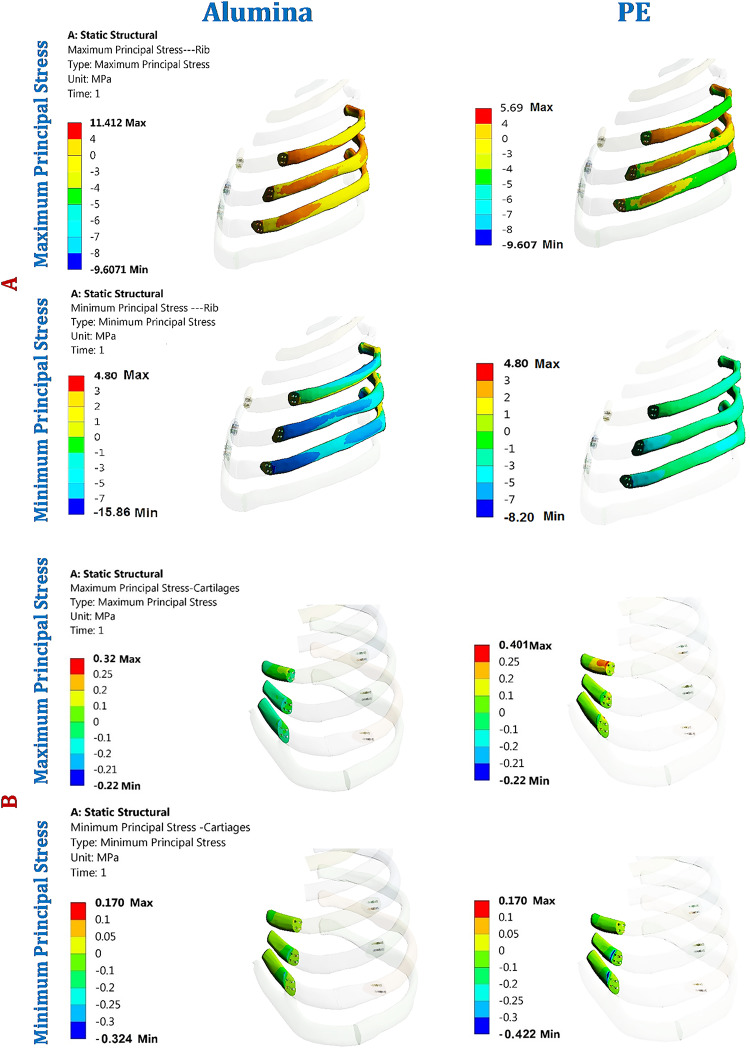
Distribution of maximum and minimum principal stresses (MPa) on: (**A**) Ribs and (**B**) Cartilages under normal breathing using alumina and PE implants.

As shown in Figs. 6 and 7, and 8, the maximum tensile and compressive stresses and strains were determined for the left fourth, fifth, and sixth ribs, as well as the connected cartilages and left lung, using various implants with different stiffness levels. These values were then compared to those of the intact model. Compared to the intact model, alumina, zirconia, and CFP 60% implants increased the stresses and strains on the ribs while decreasing them on the cartilages and lungs, in contrast to PEEK and PE implants. The CFP 30% implants distributed the stresses and strains more evenly across the ribs, cartilages, and lung, similar to the intact model. Figure 9 shows the distribution of maximum and minimum principal stresses on the ribs and cartilages during normal breathing, using stiff alumina and soft PE implants.

Figure 6 illustrates that the increase in implants stiffness slightly raises the ribs stresses and strains. When using alumina implants, the maximum tensile and compressive stresses increased by 2.79% and 1.79%, respectively. Consequently, the maximum tensile and compressive strains increased by 0.98% and 1.24%, respectively. In the case of zirconia implants, the maximum tensile and compressive stresses increased by 1.98% and 0.899%, respectively, leading to an increase in maximum tensile and compressive strains by 0.52% and 0.8%, respectively. CFP 60% implants also caused a slight increase in stresses and strains on the ribs. On the other hand, CFP30%, PEEK, and PE implants decreased the maximum tensile and compressive stresses by 11.26% and 32.92%, 21.89% and 40.43%, and 48.73% and 47.36%, respectively. Additionally, the maximum tensile and compressive strains decreased by 8.22% and 7.6%, 13.87% and 12.18%, and 15.38% and 17.66%, respectively, when using these implants.

Under normal breathing, increasing the stiffness of the implant decreased the stresses and strains on the surrounding cartilages, as shown in Fig. 7. The alumina, zirconia, and CFP 60% implants decreased the maximum tensile and compressive stresses by 19.39% and 22.48%, 16.37% and 17.46%, and 5.79% and 5.98%, respectively. This resulted in a decrease in the maximum tensile and compressive strains by 22.35% and 28.35%, 16.09% and 27.05%, and 5.83% and 7.35%, respectively. On the other hand, CFP 30%, PEEK, and PE implants increased the stresses and strains on the surrounding cartilages, but these increases did not exceed 1.5%.

In Fig. 8, it is shown that alumina and zirconia implants reduce stresses and strains on the lung by up to 1–4% compared to titanium implants. The stresses and strains on the lungs with CFP 60% and CFP 30% implants were similar to those with titanium implants. However, PEEK and PE implants increased maximum tensile and compressive stresses by 34.34% and 39.69%, as well as 72.72% and 87.39%, respectively. This led to an increase in maximum tensile and compressive strains by 33.88% and 39.96%, and 72.36% and 103.98%, respectively.

### Impact force

#### Stresses on implants

**Fig. 10:**
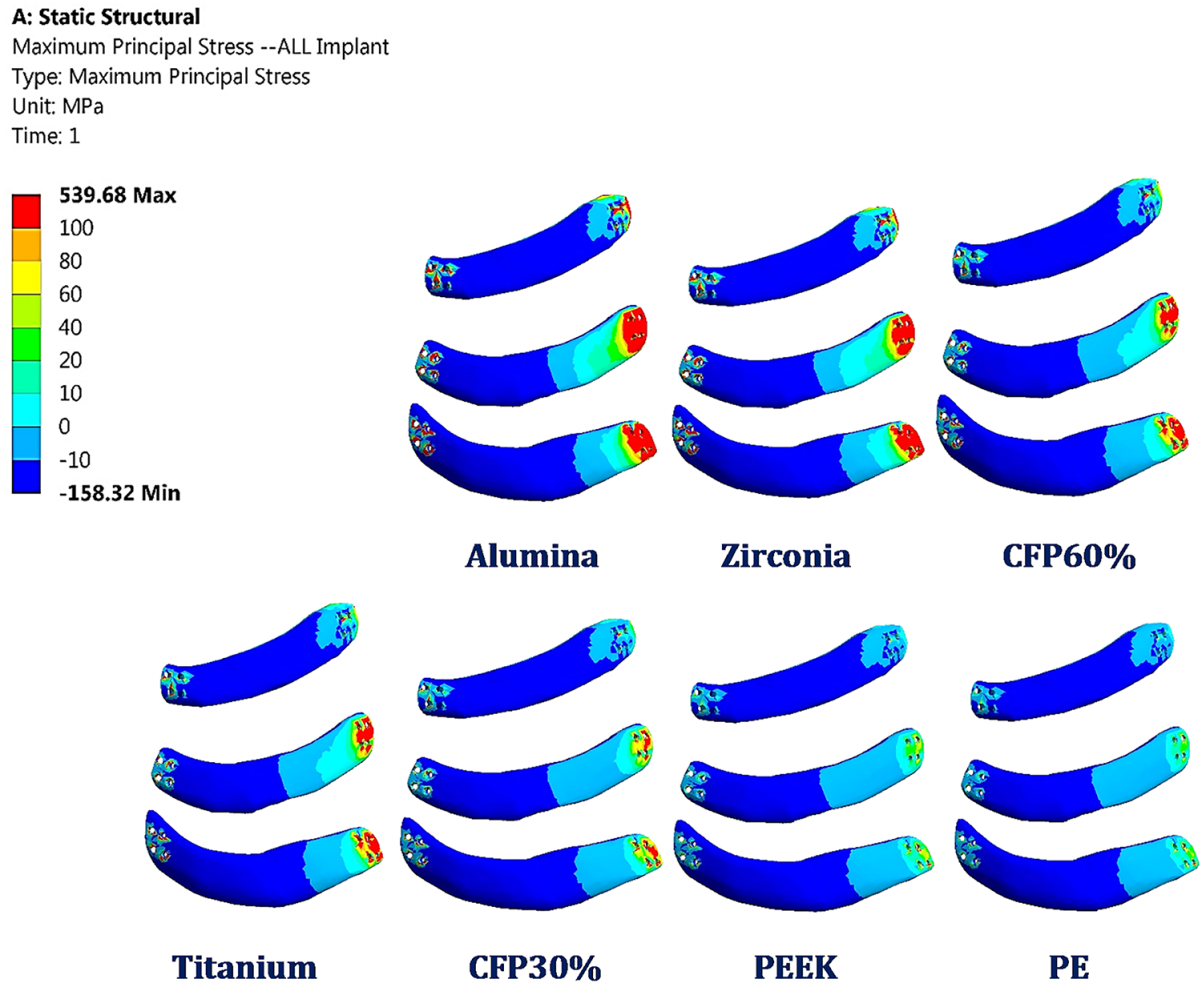
Distribution of maximum principal stresses (MPa) on the alumina, zirconia, CFP 60%, titanium, CFP 30%, PEEK and PE implants (front view) under impact force.

Under impact force, the maximum tensile and compressive stresses were calculated (see Table 4) for the implants to assess their durability. Figure 10 shows the distribution of maximum principal stresses (in MPa) on the implants under the impact force. Compared to titanium implants, the maximum tensile and compressive stresses increased by 76.6% and 101.55%, 33.99% and 44.33%, and 18.4% and 26.93% on alumina, zirconia, and CFP 60% implants, respectively. Conversely, the maximum tensile and compressive stresses decreased by 41.8% and 53.9%, 51.37% and 60.58%, and 74.96% and 82.315% on CFP 30%, PEEK, and PE implants.

**Table 4:** Maximum tensile and compressive stresses (MPa) on implants under impact force.

	AL	ZR	CFP60	TI	CFP30	PEEK	PE
Implants							
Max Tensile Stress	539.68	409.37	361.73	305.51	177.8	148.57	76.496
Max Compressive Stress	-898.33	-643.3	-565.76	-445.7	-205.4	-175.6	-78.82

#### Stresses on screws

**Fig. 11:**
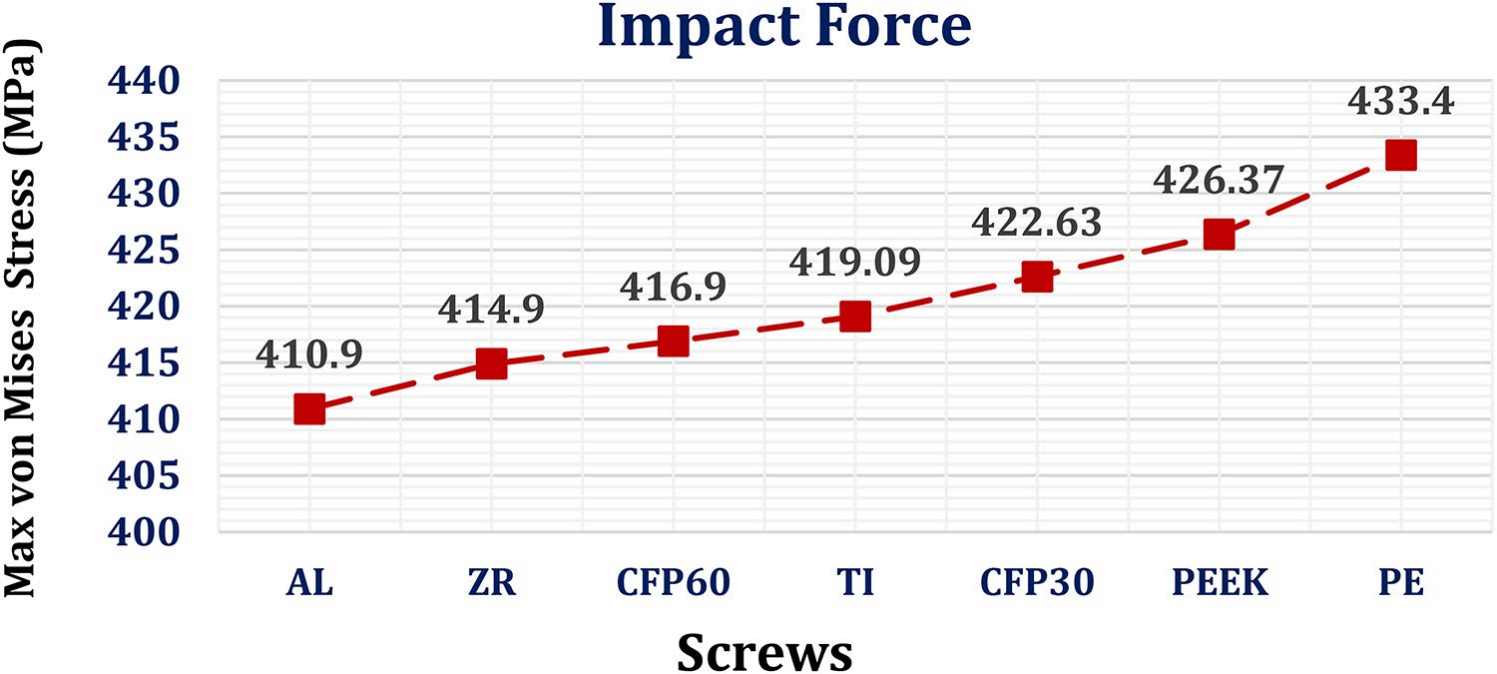
Maximum von Mises stresses (MPa) on screws, using different implants materials, under impact force.

Under the impact force, the alumina, zirconia, and CFP 60% implants decreased the maximum von Mises stress on screws by 1.95%, 1%, and 0.523%, respectively, compared to titanium implants, as shown in Fig. 11. Conversely, CFP 30%, PEEK, and PE implants increased the maximum stress on the screws by 0.845%, 1.737%, and 3.41%, respectively.

#### Stresses and strains on biological parts

**Fig. 12:**
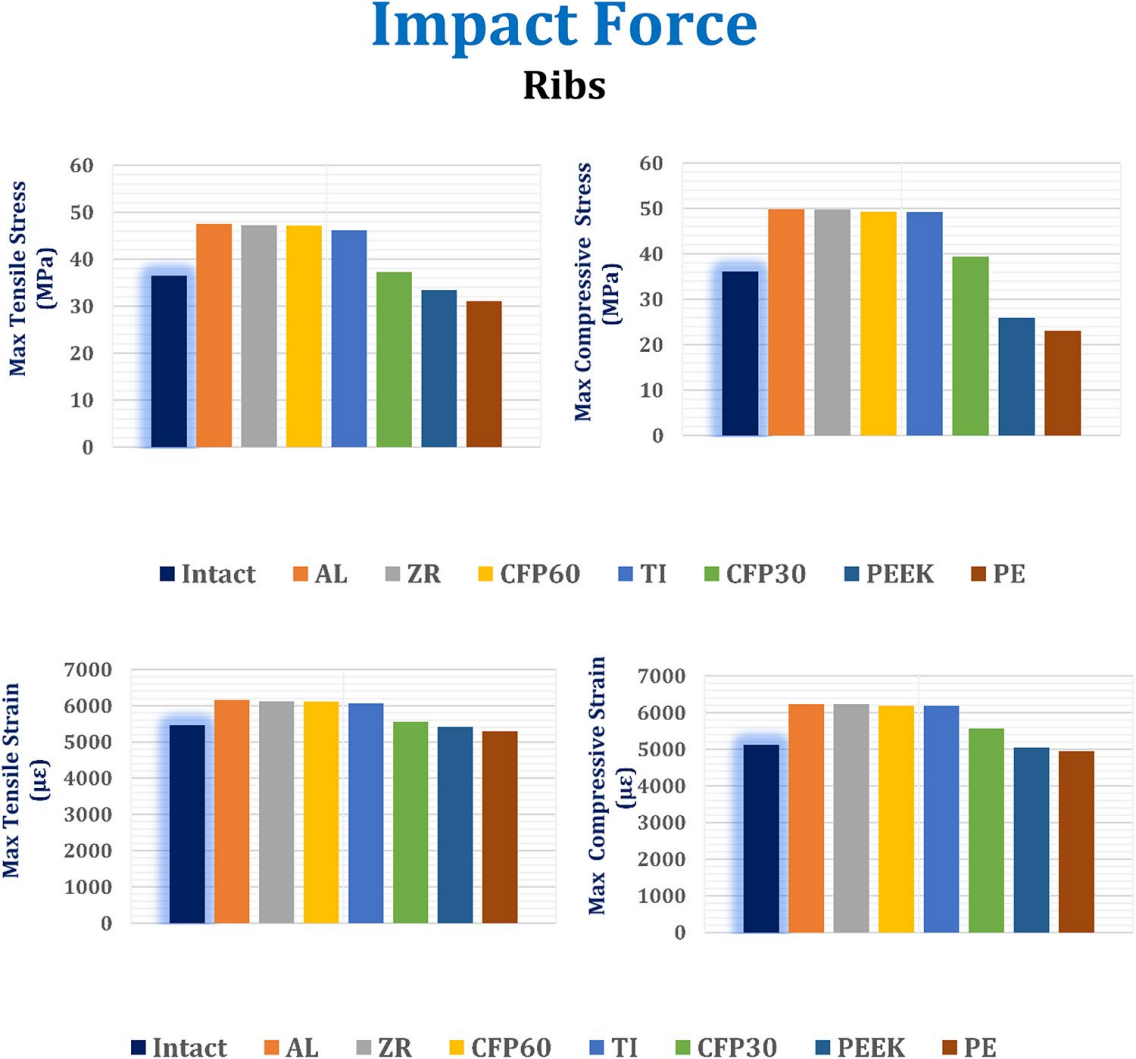
Maximum tensile and compressive stresses (MPa) and maximum tensile and compressive strains (µε) on the ribs under impact force.

**Fig. 13:**
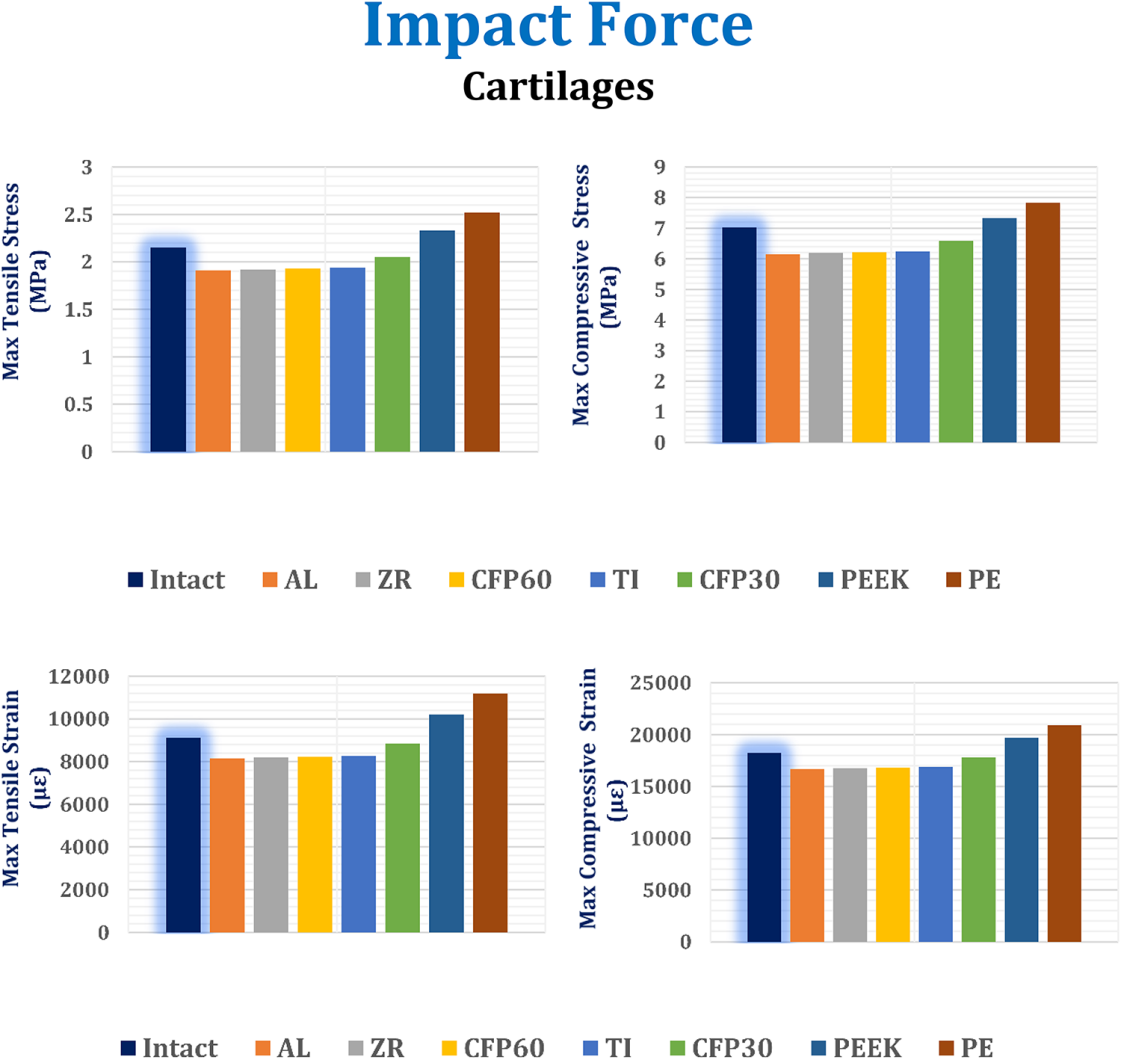
Maximum tensile and compressive stresses (MPa) and maximum tensile and compressive strains (µε) on the cartilages under impact force.

**Fig. 14:**
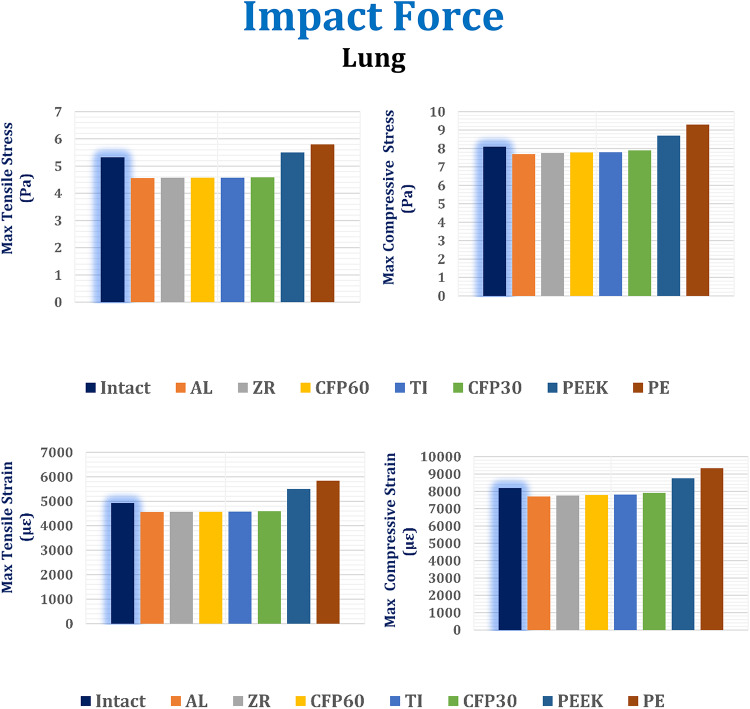
Maximum tensile and compressive stresses (MPa) and maximum tensile and compressive strains (µε) on the lung under impact force.

In this section, the maximum tensile and compressive stresses, and strains are derived (Figs. 12, 13 and 14) for the left fourth, fifth, and sixth ribs, the connected cartilages and left lung, using different implant materials. As shown in the figures, stresses and strains using alumina, zirconia, and CFP 60% implants increased on the ribs but decreased on the cartilages and lungs compared to the intact model, in contrast to PEEK and PE implants. Additionally, the CFP 30% implants distributed stresses and strains more evenly across the ribs, cartilages, and lungs, similar to the intact model.

For the ribs (Fig. 12), when alumina implants were used, the maximum tensile and compressive stresses increased slightly by 2.97% and 1.32%, respectively. As a result, the maximum tensile and compressive strains also increased by 1.6% and 0.81%, respectively. When using zirconia implants, the maximum tensile and compressive stresses increased by 2.35% and 1.12%, respectively, leading to an increase in maximum tensile and compressive strains by 0.92% and 0.71%, respectively. Compared to titanium implants, CFP 60% implants had minimal impact on the stresses or strains on the surrounding ribs. However, CFP 30%, PEEK, and PE implants decreased the maximum tensile and compressive stresses by 19.26% and 19.95%, 27.55% and 47.3%, and 32.71% and 53.08%, respectively. Additionally, the maximum tensile and compressive strains decreased by 8.42% and 10.025%, 10.62% and 18.37%, and 12.68% and 20.05%, respectively, when using these implants.

Under the impact force, as shown in Fig. 13, the alumina, zirconia, and CFP 60% implants slightly reduced the maximum tensile and compressive stresses and strains on the cartilages. However, for the CFP 30%, PEEK, and PE implants, the maximum tensile and compressive stresses increased by 5.67% and 5.6%, 20.10% and 17.46%, and 29.89% and 25.48%, respectively. Consequently, the maximum tensile and compressive strains also increased by 6.9% and 5.33%, 23.32% and 16.5%, and 35.33% and 23.82%, respectively.

As shown in Fig. 14, compared to titanium implants, alumina and zirconia implants decreased the stresses and strains on the left lung by no more than 1.5%. Additionally, CFP 60% implants distributed stress and strain to the lungs similarly to titanium implants. On the other hand, CFP 30%, PEEK, and PE implants increased the maximum tensile and compressive stresses by 0.43% and 1.28%, 20.35% and 11.53%, and 26.91% and 19.23%, respectively. Consequently, they also increased the maximum tensile and compressive strains by 0.32% and 1.3%, 20.21% and 12.06%, and 27.57% and 19.5%, respectively.

**Fig. 15:**
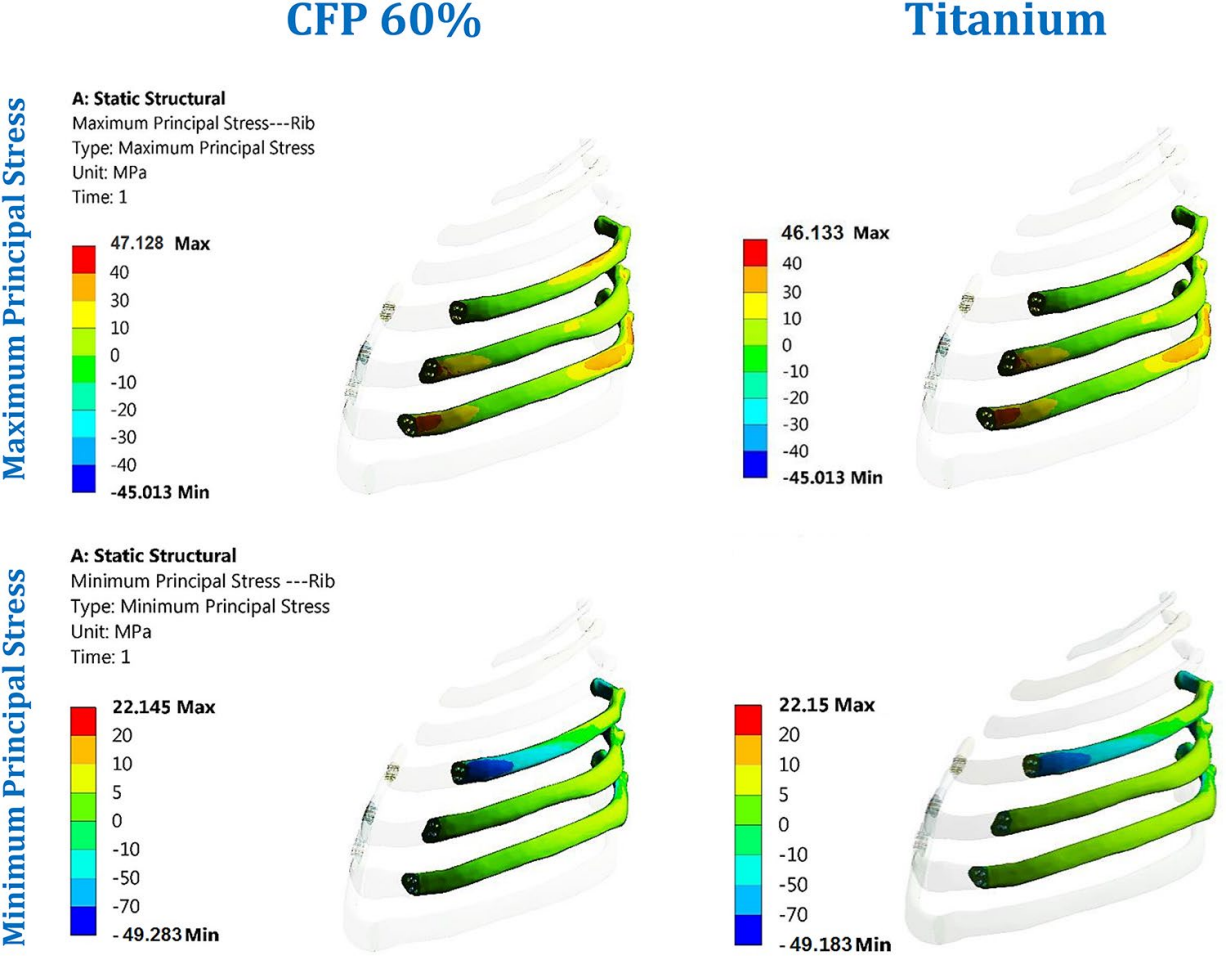
Distribution of maximum and minimum principal stresses (MPa) on the surrounding ribs under impact force using CFP 60% and titanium implants.

Figure 15 illustrates the distribution of maximum and minimum principal stresses on the left ribs and connected cartilages under impact force using CFP 60% and titanium implants. The figure demonstrates that CFP 60% implants distributed the stresses on ribs similar the titanium implants.

### Implants fatigue

**Fig. 16:**
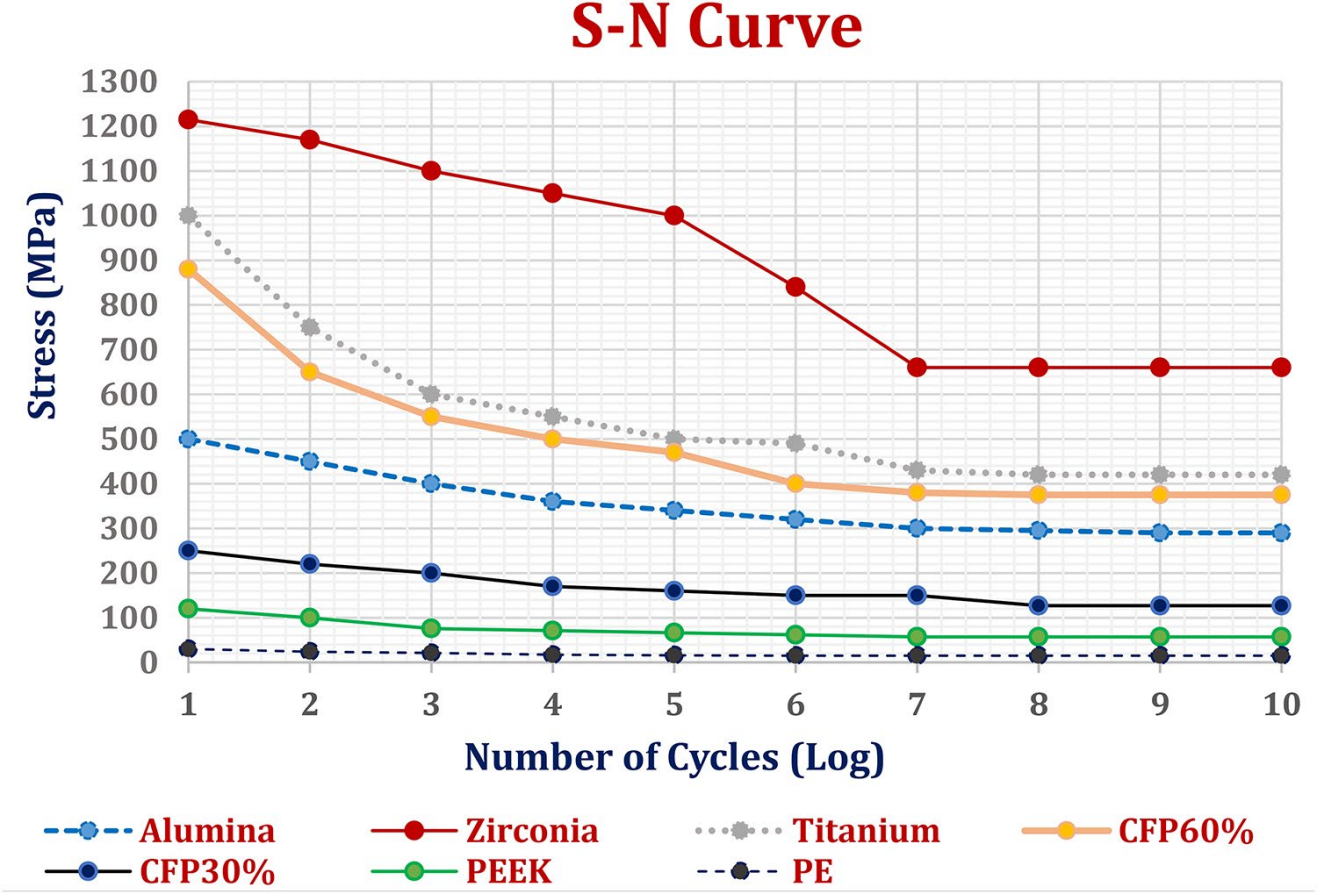
**S**-N curves of different materials^73–80^.

Fatigue analysis was conducted on three customized implants made of different materials under normal breathing conditions (see Table 5) and impact forces (see Table 6). The S-N curve (see Fig. 16) illustrates the fatigue behavior of each material, with stress on the ordinate axis and the number of cycles on the abscissa axis. Three parameters were considered for the assessment. The first parameter was the life cycle, which indicates the failure of the material due to repeated loads. The second parameter was the alternating stress, which represents the relationship between the maximum and minimum forces acting on the implants. The third parameter was the safety factor^73–80^.

Under normal breathing conditions, the life cycles of all materials were estimated to be e^10^, meaning no implant would fail. The maximum equivalent alternating stresses were 43.381, 31.287, 26.735, 22.913, 18.54, 13.442, and 8.1445 MPa for alumina, zirconia, CFP 60%, titanium, CFP 30%, PEEK, and PE implants, respectively. The fatigue limits of these materials were approximately 290, 660, 375, 420, 120, 57, and 15 MPa, resulting in safety factors with minimum values of 6.68, 15, 14, 15, 6.47, 4.2, and 1.84 for alumina, zirconia, CFP 60%, titanium, CFP 30%, PEEK, and PE implants, respectively. Additionally, all implants had a maximum safety factor of 15.

Under impact force (Table 6), alumina, PEEK, and PE implants would fail, as their maximum equivalent alternating stresses of 700.62, 146.6, and 70.29 MPa exceeded their fatigue limits of 290, 57, and 15 MPa respectively, resulting in minimum safety factors below one. The zirconia, titanium, and CFP 30% & 60% implants exhibited superior mechanical behavior compared to other implants, as expected due to their high endurance, providing a life equivalent to more than one billion load cycles. This can be confirmed by the maximum equivalent alternating stresses being lower than their fatigue limits, resulting in minimum safety factors of 1.46, 1.037, 1.44, and 1.02 for zirconia, CFP 60%, titanium, and CFP 30% respectively.

**Table 5:** Fatigue analysis of implants with different materials, under normal breathing.

		AL	ZR	CFP60	TI	CFP30	PEEK	PE
Life	Max	1e10	1e10	1e10	1e10	1e10	1e10	1e10
	Min	1e10	1e10	1e10	1e10	1e10	1e10	1e10
Safety Factor	Max	15	15	15	15	15	15	15
	Min	6.68	15	14	15	6.47	4.2	1.84
Equivalent Alternating Stress (MPa)		43.381	31.287	26.735	22.913	18.54	13.442	8.1445

**Table 6:** Fatigue analysis of implants with different materials, under impact force.

		AL	ZR	CFP60	TI	CFP30	PEEK	PE
Life	Max	1e10	1e10	1e10	1e10	1e10	1e10	1e10
	Min	0	1e10	1e10	1e10	1e10	0	0
Safety Factor	Max	15	15	15	15	15	15	15
	Min	0.413	1.46	1.037	1.44	1.02	0.388	0.213
Equivalent Alternating Stress (MPa)		700.62	449.9	361.36	289.83	117.5	146.6	70.29

**Fig. 17:**
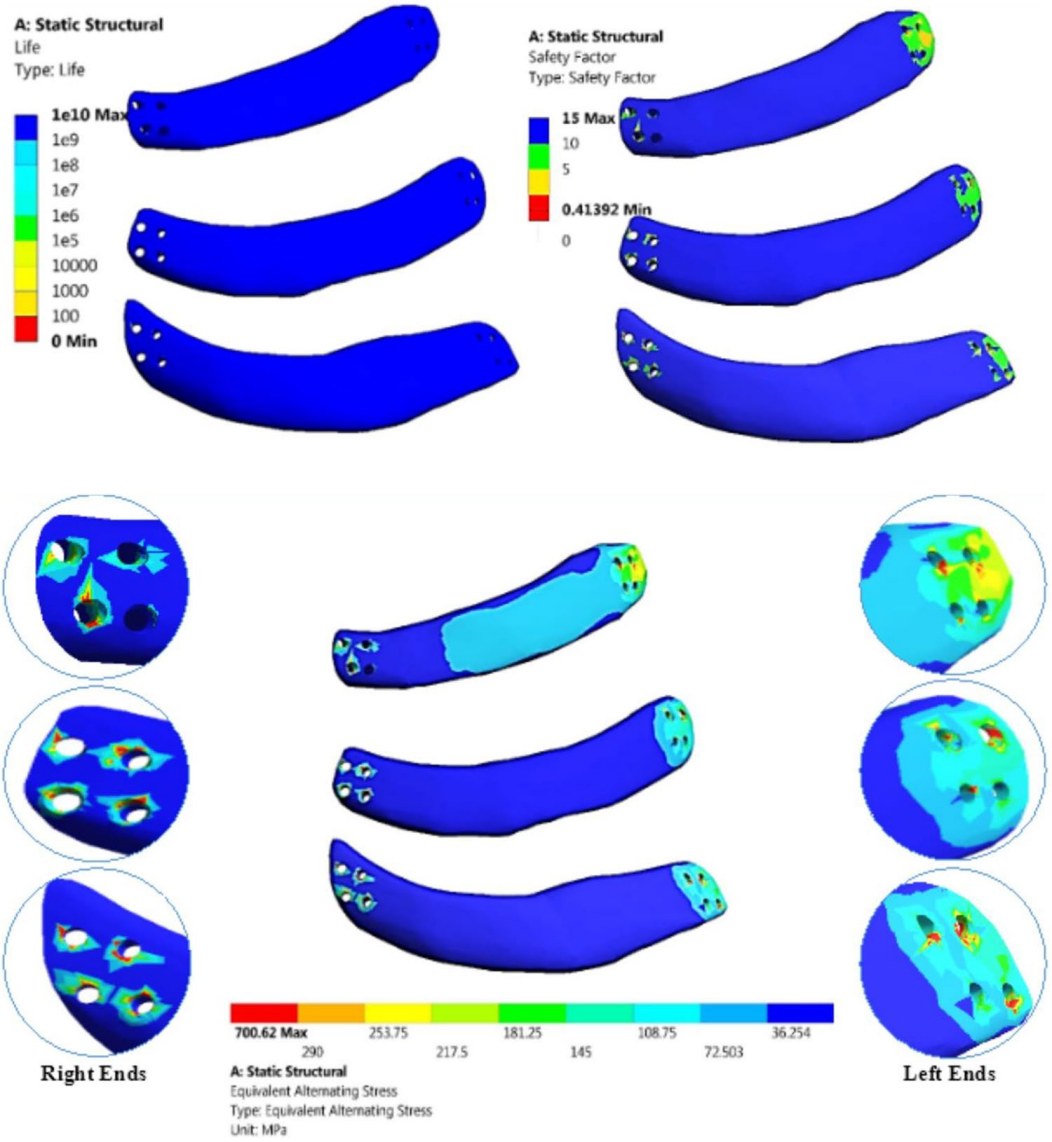
Life, safety factor and equivalent alternating stress of alumina implants, under impact force.

Figure 17 illustrates the life cycles, safety factor, and equivalent alternating stress of alumina implants under impact force. The red color in equivalent alternating stress represents the fractured region where the stresses exceed the fatigue limit of 290 MPa.

## Discussion

The most common causes of chest wall defects are excisions of a portion of the chest wall due to infection, car accidents, metastatic lesions, locally invasive tumors, and malignancies^1,3^. Lateral or infero-anterior resections, along with resections of the sternum, usually invariably necessitate prosthetic restoration for stability and coverage of critical intrathoracic structures because there is no substantial muscle coverage in these locations^81,82^. Furthermore, regardless of location, defects in the anterior chest larger than 5 cm, posterior defects larger than 10 cm, or a resection involving more than 3 ribs usually necessitate skeletal restoration using a biological, alloplastic, or synthetic material^83^.

The finite element method is useful for evaluating the biomechanics of respiration and their effects on the rib cage, treatment planning of chest wall defects, designing implants and instruments, simulating various materials under different circumstances, and extracting internal stresses and strains at each point^34,68^. In Roshan et al.^68^, finite element analysis was used on a rib cage model to identify stress distributions and calculate the rate of bone fractures, particularly in pathologically altered bones. Additionally, the analysis aimed to calculate the load and stress that an accident would exert on the human rib cage. In the static analysis, the front part of the rib cage was subjected to loads of 400, 600, and 1000 N, and the maximum stress and deformation were recorded at each load. The results showed that the maximum stress values on the rib cage were 10.33, 15.537, and 39.183 MPa under 400 N, 600 N, and 1000 N, respectively. These values fell within acceptable limits, indicating that no failure would occur.

The process of reconstructing the chest wall involves restoring its skeletal stability and stiffness, preserving breathing mechanics, safeguarding the intrathoracic organs, and reducing the risk of local and systemic postoperative complications^4^. Chest repair using customized plates and implants has been a common surgical procedure for fixing chest wall defects; however, this repair remains debatable regarding the procedure and the type of material or implant to be used^84^. In addition, the location and the size of the defect that needs to be reconstructed are principal factors to consider^85^. The development of Additive Manufacturing (AM) technology has made it possible to produce custom-made implants for chest wall reconstructions. Based on the exact location and dimensions of the chest wall resection, an implant model that fully fulfills the patient’s anatomy can be created using the patient’s computed tomography (CT) data^86,87^.

Rib prostheses have been made using a wide range of materials, including bioprosthetic materials like pig dermis, AlloDerm, and bovine pericardium, as well as synthetic alloplastic and metallic materials^9^. Additionally, with the advancement of three-dimensional (3D) printing technology, custom-designed titanium or alloplastic implants have become popular for their excellent anatomical reconstruction^88–90^.

Metallic materials such as pure titanium, cobalt-based alloys, stainless steel, and titanium alloys (e.g., grade 5) are frequently used in the fabrication of implants due to their durability, compatibility, and stability^8,9^. Demondion et al.^87^ fabricated a Ti-6Al-4 V sternum after subtotal sternotomy for an isolated BC metastasis. The artificial part consisted of a plate fastened to three staples on each side, which were then tightened on multiple ribs. At a 6-month follow-up, the patient reported no chest pain, dyspnea, or difficulty in performing daily activities.

In the report by Turna et al.^91^, a large anterior chest wall defect was reconstructed using a patient-specific custom-made titanium implant. The titanium implant was placed after the resection of an infected mammary tumor recurrence in a 62-year-old female patient. The tumor was located in the anterior chest wall, including the sternum. A latissimus dorsi flap and split-thickness graft were also used to successfully cover the implant. The report demonstrated that a custom-made titanium chest wall implant could be a viable alternative for patients with large chest wall tumors.In a study by Wen et al.^46^, a customized titanium implant created through rapid prototyping and 3D printing was used for chest wall reconstruction. ANSYS software was utilized to analyze the mechanical properties and performance of the produced implants. The results indicated that using a titanium implant in the reconstruction process was a reliable method for correcting bone defects.

In the research conducted by Jiang et al.^52^, five face-centered cubic (FCC) stainless steel lattice constructs were utilized as rib implants. Stainless steel offers several advantages due to its availability, affordability, and resistance to corrosion in various environments. However, bulk SS316L has a significantly higher density and stiffness compared to rib bone tissue, which could potentially lead to negative outcomes such as bone weakening^92^. Lattice structures have emerged as promising options for metallic implants due to their lightweight nature and favorable mechanical properties. By carefully designing and constructing these structures, it is possible to minimize the density and stiffness mismatch between SS316L and its application as a rib implant. The study’s findings indicated that the FCC-XYZ lattice design, created using a laser energy density parameter of 125 J/mm3, could be utilized for producing customized implants.

Metals offer numerous advantages in the chest wall repair process, but due to their drawbacks, they may not always be suitable^10,11^. Some disadvantages of metals include the risk of inducing allergies, hypersensitivity reactions, the need for surface modifications, casting challenges, incompatibility with CT and MRI imaging systems, and aesthetic concerns. Furthermore, they have been associated with various clinical problems, such as contamination and surface deterioration linked to peri-implantitis^12^.

Recently, polymeric materials such as polymethylmethacrylate (PMMA) and ultra-high molecular weight (UHMW) have been utilized to reconstruct large chest wall defects following the resection of chest wall tumors^9,93^. The biomechanical behaviors of different prosthetic materials (steel, titanium, and PMMA) under conditions of critical rib fracture were analyzed using the finite element method^63^. The three prosthetic materials were tested under three load conditions: sternal load (an anterior-posterior load applied at the third rib), lateral load (strength applied at the lateral arch of the fifth rib), and vertical load (vertical load applied at the first sternocostal junction).In terms of failures, the results showed that the polymethylmethacrylate prosthesis produced results identical to those obtained from the simulation using the finite element method and can be used in chest wall repair^63^.Titanium and UHMW were used as implant materials in Magesh’s research^94^. The results showed that the UHMW implants did not perform as well as the titanium implants. However, both types of implants were found to be suitable for reconstructing the chest wall.

Recently, new polymeric materials, such as PEEK (polyether ketone) and polyethylene (PE), have been scientifically confirmed to be safe for use in orthopedic fixation and reconstruction^20,21^. Polyether ketone (PEEK) and polyethylene (PE) are soft polymeric materials that have been added to the list of materials suitable for use in orthopedics and dentistry for making screws, plates, bridges, implants, and abutments. These materials can distribute the load uniformly and reduce the stresses transferred to the bone and substructure components due to their low Young’s modulus and superior shock absorption capacity compared to metals^25,26^.

Kang et al.^95^ developed a unique method for creating a customized PEEK rib prosthesis that was best suited for the FDM manufacturing process. The findings suggested that the centroid trajectory derived from a natural rib could provide reliable guidance for rib prosthesis design. Furthermore, the PEEK rib prosthesis exhibited positive clinical results and was implanted effectively. From the results extraction and evaluation, the values of maximum von Mises stress on C-design and D-design models were 67.67 and 69.98 MPa, respectively. These values did not exceed the critical limit of 88 MPa, indicating the feasibility of using a PEEK rib prosthesis.

The goal of the case report by Lipińska et al.^31^. was to introduce a unique technique for reconstructing the manubrium after excision due to cancer using a custom-designed polyethylene implant. The findings demonstrated that, in comparison to previous techniques of filling defects following the excision of a tumor in this region, specially designed sternal implants made using 3D technology represent an intriguing option.

PEEK composites, such as carbon fiber-reinforced PEEK at 30% and 60%, have recently been used for bone fixation and reconstruction. The exceptional mechanical qualities, biocompatibility, adaptability, and compatibility of these composites with imaging technology have made them very appealing for the fabrication of dental and orthopedic implants. These composites have a wide range of mechanical, surface, and physical properties and can be fabricated in different shapes^26,28,29^.

In the study by Zhang et al.^38^, clinical application and finite element analysis were used to evaluate the biomechanics of carbon fiber artificial ribs. The results showed that, in a frontal collision simulation of the artificial rib overall chest model, the highest stress reached 40.58 MPa at the rib’s end. In the simulation results of the artificial bone model after impact, the overall chest stress reached 13.11 MPa. Based on these findings, the clinical outcomes and integrated simulations confirmed that carbon fiber artificial ribs for chest wall reconstruction have good mechanical performance and are biocompatible under both static and dynamic stress while maintaining normal respiratory function.

In contrast to polymeric materials, some researchers have recommended using stiff bioceramics, such as zirconia or alumina, in the fabrication of implants instead of titanium^12–15^. These bioceramics have been frequently utilized in dentistry and orthopedics for bone fixation and reconstruction due to their good mechanical properties, low thermal conductivity, low plaque affinity, excellent corrosion resistance, biocompatibility, and superior aesthetics^14–16^. Moreover, they can be used for load-bearing applications in bone repair and in the area of femoral heads for total hip replacements^17^. Considering their physical properties, Sampaio et al.^96^ reported that porous zirconia blocks could be used as substitutes for bone graft materials for restoring extensive bone sites. In dentistry, zirconia has been characterized as a dental biomaterial for fabricating dental bridges, screws, crowns, inserts, and implants, mostly because of its biocompatibility, high fracture toughness, and radiopacity^97^.

In the research by Bertin et al.^98^, a porous alumina prosthesis was reconstructed for sternal replacement. It possessed interesting characteristics such as great biocompatibility, a high level of bacterial resistance, radiolucency, and compatibility with radiotherapy. The implant was stitched to the ribs using suture thread without the need for osteosynthesis material. Six patients, with a mean age of 60.6 years, received this prosthesis and were followed up for an average of 20 months. No major complications, breathing issues, or pain related to the prosthesis occurred in any of the patients.

The goal of the current study was to evaluate the strength of chest wall prostheses used for reconstructing the resected portions of ribs and cartilages, while also analyzing the stress and strain on the lungs, cartilage, and ribs using two distinct loading scenarios. Firstly, the model was validated with the intact model of the rib cage constructed by Suazo et al.^72^. Cardiopulmonary resuscitation maneuvers were simulated by five compression areas (P1, P2, P3, P4, and P5) with areas of 10 cm^2^. For validation, the maximum compression depth (cm) and the maximum von Mises stresses (MPa) were extracted. Compared with Suazo et al.^72^ results, there were slight differences between the results (not exceeding 6%).

After validation, the process of chest wall reconstruction was carried out in the resected portions of ribs and cartilage using three custom-made implants. The materials used for the implants were stiff bioceramics (alumina and zirconia), soft polymers (polyether ether ketone (PEEK) and polyethylene (PE)), and polymeric composites (carbon fiber-reinforced PEEK 30 and 60% (CFP 30 and 60%)). To assess the outcomes, the maximum tensile and compressive stresses were used to evaluate implants made of different materials. Additionally, the maximum tensile and compressive stresses, as well as strains, were calculated for the lungs, cartilage, and ribs. The null hypothesis, predicting that stiff implants would provide an optimal scenario when used in the reconstruction of both ribs and cartilage, was partially accepted.

The findings from static analysis showed that stiff bioceramic implants (alumina and zirconia) resulted in the lowest stresses and strains on the cartilages and lung, but the highest stresses and strains on the ribs. However, the increase in rib stresses and strains did not exceed 3%. In contrast, soft PEEK and PE implants caused the highest stresses and strains on the cartilages and lung, while producing the lowest stresses and strains on the ribs. Additionally, the results indicated that the stresses on the ribs, cartilage, and lungs of the CFP 30% and CFP 60% implants were similar to those on the titanium implants.

**Fig. 18:**
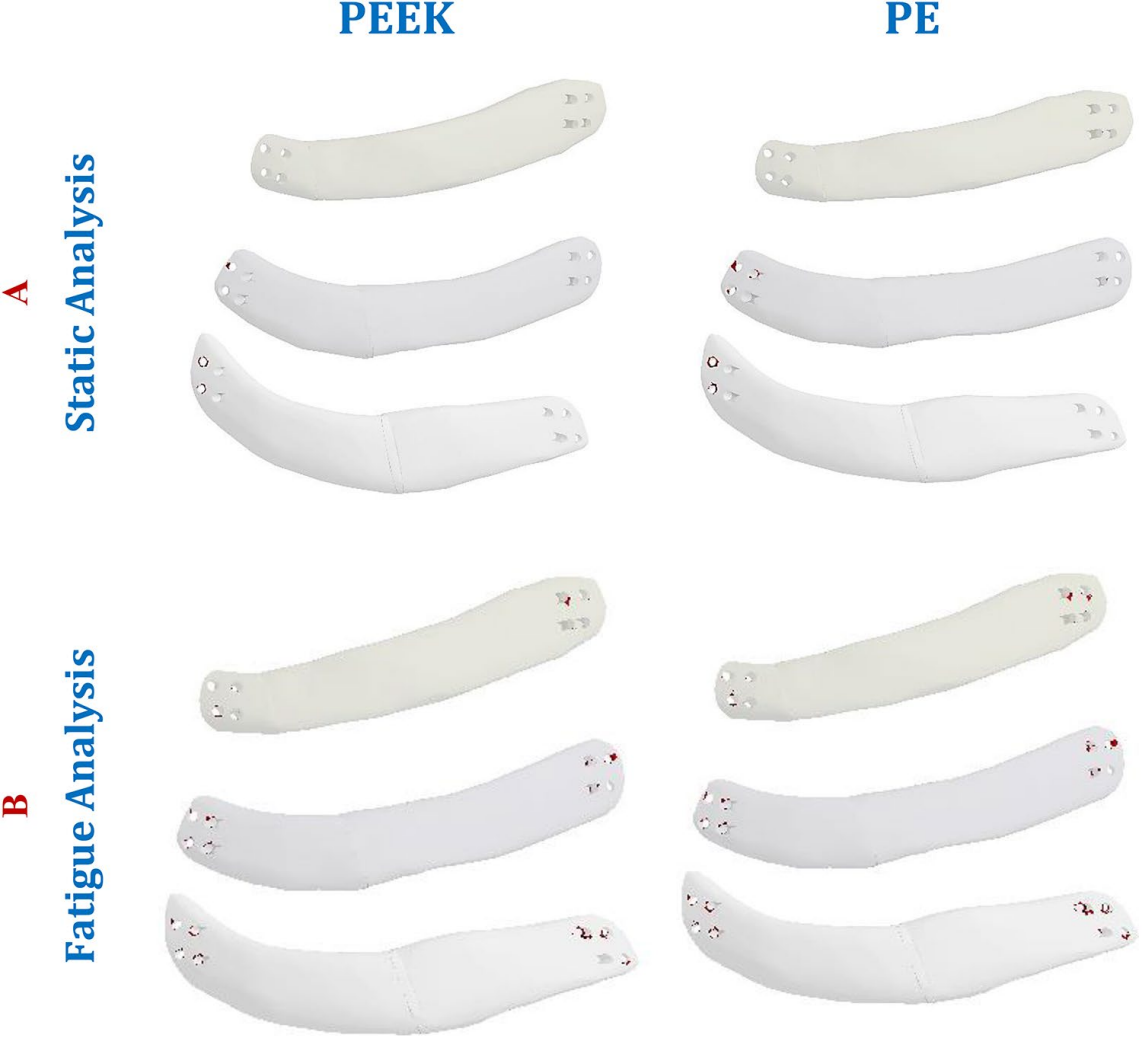
Expected fracture areas (red) in PEEK and PE implants, under an impact force of 1000 N: (**A**) Static analysis, and (**B**) Fatigue analysis.

In the static investigation of the implants, the maximum tensile and compressive stresses were extracted and compared with the permissible limits (Table 1) under the two loading scenarios. The findings showed that soft implants (PEEK & PE) were not recommended for use because they were prone to fracture under impact force. Figure 18 illustrates the expected fractured areas (highlighted in red) of the PEEK and PE implants under an impact force of 1000 N. Although alumina is a strong bioceramic, it may be damaged under the influence of impact force, as the maximum tensile stress exceeded the limit of 275 MPa. For screws, no failure was expected as the maximum von Mises stresses did not exceed the titanium yield limit.

A fatigue study was conducted on implants made of different materials. The results showed that during normal breathing, all materials had a life cycle of at least 10^6^ cycles and safety factors above one, indicating that no implants would fail. When subjected to impact force, zirconia, titanium, and CFP30& 60% implants exhibited superior mechanical behavior compared to other implants, with a life equivalent to over one billion cycles. Additionally, their maximum equivalent alternating stresses were below their fatigue limits, resulting in a minimum safety factor of more than one. For alumina, PEEK, and PE implants, failure was anticipated at the right and left ends where they came into contact with screws (see Fig. 18).

For biological tissues such as ribs, cartilage, and lungs, the maximum tensile and compressive stresses and strains were calculated and compared to the tensile and compressive limits (show to Table 1 and the “Results Extraction” section). While the stress and strain on the lung and cartilages were significantly higher with PEEK and PE implants compared to other implants, these values still fell within acceptable limits. Similarly, although alumina and zirconia implants increased the stresses and strains on the ribs, these increases were less than 3% and did not surpass the allowable limits.

**Fig. 19:**
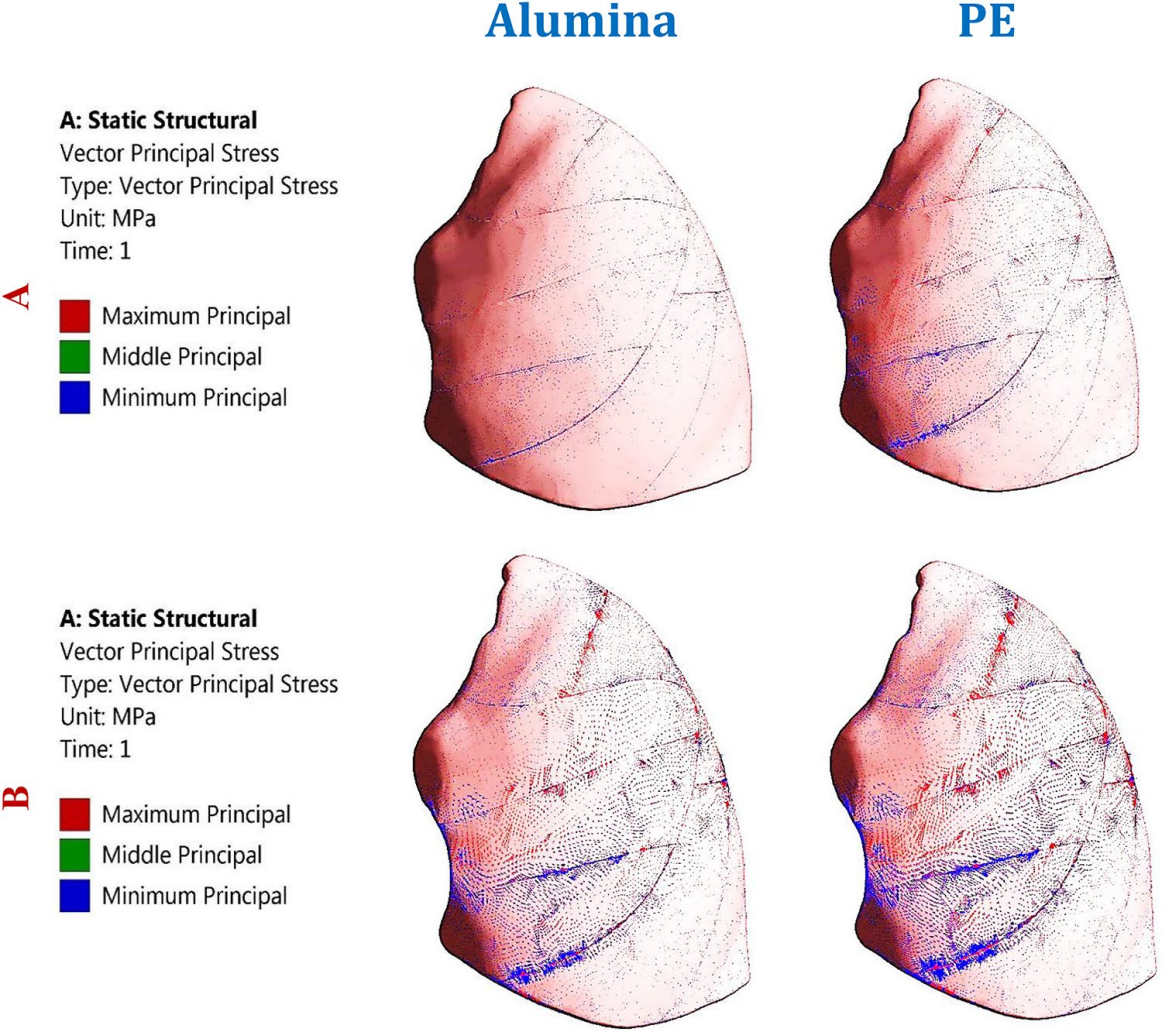
Principal stress vectors of the left lung using alumina and PE implants, under: (A) Normal breathing, and (B) Impact force. Red arrows (tension) and blue arrows (compression).

Figure 19 shows the principal stress vectors of the left lung using alumina (highest stiffness) and PE (lowest stiffness) implants. The areas of tension are indicated by red arrows, while compression is shown with blue arrows, under impact force. The figure demonstrates that PE implants concentrated the tensile and compressive stresses more than alumina implants. Additionally, the impact force significantly affected the lung, potentially causing damage with repeated force.

Finally, it was concluded that in the chest wall reconstruction process, semi-stiff implants (CFP 30%) and stiff implants (zirconia and CFP 60%) can be utilized as titanium alternatives to reconstruct the defective portions of cartilages and ribs. These implants are characterized by excellent properties and compatibility and are not linked to clinical issues. In addition, they are safe for surrounding structures, do not cause inflammation, and are not subjected to deformation or fracture under sudden impact forces. Furthermore, CFP 30% and CFP 60% composites have a wide range of mechanical, surface, and physical properties and can be fabricated in different shapes. Three key benefits arise from using these polymeric composites: improved performance, reduced total system cost, and expanded design freedom^26–29^.

The limitations of this study included the assumption that the biological materials (ribs, cartilage, and lungs) were homogenous and isotropic. It is important to note this limitation when interpreting the results, as bone and lungs are more complex and anisotropic^99–101^. Additionally, full osseointegration of the implants with the surrounding ribs and cartilages was expected, but may not be achievable in actual clinical settings. Other limitations revolved around the design and placement of the implants, as any adjustments could impact the outcomes. Furthermore, the method for determining the load and boundary conditions was theoretical and may not accurately reflect real-world clinical scenarios.

Further experimental and surgical studies, as well as dynamic finite element investigations (such as breathing during sleep in worst-case postures), are necessary in the future to explore other cases of chest wall defects.

## Conclusion

The purpose of this research was to develop a reconstruction process for ribs and cartilages using artificial implants made of different materials instead of metals, and to analyze their mechanical performance using the finite element method (FEM). The materials used for the implants were alumina, zirconia, PEEK, polyethylene PE, and carbon fiber-reinforced PEEK (30% and 60%). Stress evaluations were conducted on the implants, screws, ribs, cartilages, and lungs under two different loading scenarios: normal breathing and exposure to an impact force from an accident following chest wall reconstruction. Based on the results and within the limitations of the current study, the following conclusions were drawn:

1. Stiff alumina and zirconia implants produced the least stresses and strains on the surrounding cartilages and lungs, and the highest stresses and strains on the surrounding ribs.
2. Soft PEEK and PE implants produced the highest stresses and strains on the surrounding cartilages and lungs, and the lowest stresses and strains on the surrounding ribs.
3. The stresses distributed in the ribs, cartilages, and lungs using carbon fiber-reinforced PEEK (30% and 60%) implants were similar to those using titanium implants.
4. Under static analysis, alumina, PEEK, and PE implants were susceptible to fracture as the generated tensile stresses exceeded their tensile yield strengths.
5. In the fatigue study, under normal breathing conditions, all materials had life cycles of at least 10^10^, with safety factors above one, indicating that no implant would fail.
6. In the fatigue study, under impact force, alumina, PEEK, and PE implants failed as the maximum equivalent alternating stresses exceeded their fatigue limits in specific regions (e.g., ends of implants), resulting in safety factors of less than one.
7. By utilizing all implants, the tensile and compressive stresses and strains on the ribs, cartilages, and lung were within the allowable limits.

## Data Availability

The datasets generated during and/or analysed during the current study are available from the corresponding author upon reasonable request.
